# Liquid crystal-like self-organization of glioblastoma is associated with consistent migration for high cell densities

**DOI:** 10.1038/s41598-026-58846-8

**Published:** 2026-06-22

**Authors:** Urszula Hohmann, Chalid Ghadban, Julian Prell, Christian Strauss, Faramarz Dehghani, Tim Hohmann

**Affiliations:** 1 Department of Anatomy and Cell Biology, Medical Faculty, Martin Luther University Halle-Wittenberg, 06108 Halle (Saale), Germany; 2 Department of Neurosurgery, Medical Faculty, Martin Luther University Halle-Wittenberg, 06120 Halle (Saale), Germany; 3 Department of Anatomy and Cell Biology, Medical Faculty, Martin Luther University Halle-Wittenberg, Grosse Steinstrasse 52, 06108 Halle (Saale), Germany

**Keywords:** Cell migration, Glioblastoma, Liquid crystals, Cell density, Oncostreams, Proliferation, Collective migration, Cancer, Cell biology, Neuroscience

## Abstract

Glioblastoma is the most lethal and frequent type of primary brain tumors, characterized by a high proliferative and infiltrative capacity. Here, we used live cell imaging to analyze the effect of cell density variations on the migratory capacity of established and primary glioblastoma cell lines. We found that proliferation events promoted local velocity of glioblastoma cells, up to three cell-length away from the proliferation event. Furthermore, two phenotypes were found when subjecting glioblastoma cells to a cell density gradient. While one phenotype was characterized by the active migration of cells, independent of proliferation, the other was mostly driven by cell proliferation. Lastly, the analysis of the effects of an overall increasing cell density demonstrated that cells showing signs of self-organization, forming liquid crystal-like structures are able to maintain a high migratory potential even for high cell densities. Notably, the emergence of small-scale liquid crystal-like order was associated with a better maintenance of cellular migration, even in cell populations that were largely in a state of migratory arrest. Thus, structure formation might help glioblastoma cells to move efficiently in states of high confinement, thereby maintaining infiltrative properties.

**Supplementary Information:**

The online version contains supplementary material available at 10.1038/s41598-026-58846-8.

## Introduction

Glioblastoma (GB) is an aggressive tumor, with a median survival of approximately 14 months^1^. GB is characterized by a strong proliferation and infiltration into the brain tissue, governed by the migratory properties of GB cells, moving as single cells or groups of cells along pre-existing structures, such as vessels, nerve fiber tracts, etc^2,3^. While GB cells possess large intrinsic migratory properties determined by cytoskeletal remodeling, cell-cell and cell-matrix adhesion, etc., there might be additional driving factors influencing migration, such as cell proliferation, pattern formation and cell density.

Cells, including GB cells, can undergo a transition from a fluid-like, unjammed state with high mobility into an solid-like, jammed state, blocking cellular mobility^4–13^. Modulations of cell-cell and cell-matrix adhesion, tension or increasing cell density were proposed to induce jamming^4–13^. Previous studies suggested that increasing cell density regulates traction forces, leading to migratory arrest for high cell densities^10,13^. Elucidating the role of cell density in migration is crucial, as uncontrolled proliferation and increasing cell density are universal processes in tumors. The universal cause for increasing cell density is proliferation, but how changes in cell density affect cellular migration is not fully understood^6,9,12,14–16^.

Proliferation events were identified as crucial for persistent cell mobility under confinement^17–21^. When entering mitosis, cells detach from the extra-cellular matrix, round up, build the mitotic spindle and divide^22–24^. The rounding process depends on a decrease in cell-cell and cell-substrate adhesion, and increasing intracellular pressure^25,26^, locally generating forces^27^. In endothelial cells, division events serve as sources of active stress, generating vortex-like flow patterns^18,28^ of several cell diameters in size^29^. In some models, a decline in proliferation rate was found to be responsible for a glass transition, resulting in migratory arrest. Thus, proliferation events were main drivers of cellular reorganization^17,27,30^.

Our earlier study in GB identified cell density as a crucial parameter inducing a cell-type dependent migratory arrest, manifesting as a jamming phenomenon; however, the underlying basis of this cell-type specificity remained unclear^12^. A follow-up study showed signs of self-organization in highly motile GB cells, akin to “oncostreams”^31^. Oncostreams are self-organized structures, emerging in glioma in vivo and in vitro in which cells align parallel to each other and move in parallel or anti-parallel streams^32–34^. Oncostreams were correlated with the grade and migratory capacity of gliomas^34^ and later found to be reminiscent of liquid crystal-like or nematic structures, including the presence of topological defects, corresponding to points of symmetry breaking^35^. Liquid crystal-like organization was not only observed in GB, but also in breast cancer tissue sections and was related to the migration of cell clusters^36^. Furthermore, liquid crystal-like organization was demonstrated to play a role in the maintenance and development of tissues^37^. Given the high cell density of GB in vivo, such forms of self-organization might promote an escape from the cell-density mediated migratory arrest. This idea was supported by theoretical work and experimental studies showing cell density as a main inducer of oncostreams, but they can be abrogated by disturbing e.g. calcium signaling or actin-cytoskeletal organization^32,33^. Yet, the interplay of cell density, oncostreams and self-organization in migration of GB cells has only been addressed sparsely.

The present study was designed to understand the previously identified heterogeneous response of different GB cell lines to a changing cell density that allowed certain cell types to remain motile for high cell densities. Therefore, the effect of different types of cell density fluctuations in migration was studied, such as individual proliferation events and variations in global cell density. Furthermore, the interplay of self-organization and migration was assessed as a potential escape mechanism of the cell density induced migratory arrest.

## Methods

### Cell culture

For experiments, LN229 and U138 GB cells and primary GB lines (GBM4, GBM10) were used. LN229 cells were purchased from the American Type Culture Collection (ATCC, CRL-261, Manassas, VA, USA) and U138 cells were obtained from Cell Lines Service (Cell Lines Service, 300363, Eppelheim, Germany). LN229 cells were cultured using 89% (v/v) Roswell Park Memorial Institute medium (Lonza, Basel, Switzerland, BE12-115 F), supplemented with 10% (v/v) fetal bovine serum (FBS, Gibco, Carlsbad, CA, USA, 10500-064) and 1% (v/v) penicillin/streptomycin (P/S, Gibco, Carlsbad, CA, USA, 15140-122). U138, GBM4 and GBM10 were cultured in 89% (v/v) Dulbecco’s Modified Eagle Medium (Invitrogen, Waltham, MA, USA, 41965-062), and supplemented with 10% (v/v) FBS and 1% (v/v) P/S.

Primary GB were derived from human biopsies as reported earlier^38^. All patients provided written informed consent. The study was conducted in accordance with the Declaration of Helsinki and was approved by the local Ethics Committee of the Martin Luther University Halle-Wittenberg (project reference number: 2015 − 144).

For experiments involving mitomycin C treatment to inhibit proliferation, cells were subjected to a non-lethal dose of the substance, corresponding to 0.1 µg/ml and were continuously incubated and imaged with mitomycin C^39^.

### Single cell migration

For measuring and analyzing single cell migration 1,000 cells were seeded per well in untreated 12-well plates (Greiner, Kremsmünster, Austria) 24 h prior to the start of experiments. Individual cells were imaged every 15 min with a microscope (Leica DMi8, Leica, Wetzlar, Germany) equipped with CO_2_ (5% (v/v)) and temperature (37 °C) regulation. The average cell speed and directionality were calculated based on cell tracking as described before using the Sobel operator for edge detection^40^. The directionality was defined as the ratio of the total distance travelled and the sum of incremental distances the cell moved between successive frames.

### Collective cell migration

For collective cell migration experiments 450,000 cells were seeded per well in untreated 12-well plates. Twenty-four hours afterwards cells were imaged with an inverted microscope (DMi 8, Leica, Wetzlar, Germany), in a fully humidified atmosphere with 5% CO_2_ (v/v) and at 37 °C. Images were acquired every 3 min for 60 h. For analyzing cell migration particle image velocimetry (PIV), based on PIVlab, was used with a final cross-correlation window size of 16 × 16 pixels (pixel size: 0.48 μm), as described elsewhere, to obtain local velocity fields directly from phase contrast images^12,31,41^. Briefly, PIV is a pattern matching technique, which divides an image into patches and identifies the most similar image parts in the subsequent image using cross correlation. The displacement of each patch from the current to the following image is used to estimate the local velocity.

To assess the effective motion of cells, the mean squared displacement (MSD) $\: < \left| {\Delta \:\overrightarrow {{x\left( {\Delta t} \right)}} } \right|^{2} >$ and its scaling coefficient *α(Δt)* were evaluated, using the following equation:

$$\: < \left| {\Delta \overrightarrow {{x\left( {\Delta t} \right)}} } \right|^{2} > = K\,\,{\text{* }}\Delta t^{{\alpha \:\left( {\Delta t} \right)}}$$

With the generalized diffusion coefficient *K*. The scaling coefficient retrieves information about the type of motion observed inside the monolayer, with sub-diffusive motion for *α < 1*, diffusive motion for *α ≈ 1* and super-diffuse motion for *α > 1*.

### Identifying proliferation events and cell density dependent migratory properties

To automatically identify individual proliferation events in the confluent layer, a machine learning model was used, as described previously^31^. Briefly, individual proliferation events in a dense monolayer were identified using a three-step process, consisting of an initial U-Net based segmentation, to reduce the region of interest and speed up evaluation, followed by candidate extraction using 2D cross-correlation. Finally, candidate divisions were classified as true or false positives using a GoogLeNet classifier. To match division events occurring over multiple frames the Munkres global nearest neighbor algorithm was used for detection-to-track-assignment. As cell divisions can be incomplete or cells can be excluded from the layer, having a similar appearance as mitotically rounded cells, but with a significantly longer visibility, detections that were present for more than 60 min were discarded.

To assess the influence of individual proliferation events on the layer velocity, for each identified proliferation event the speed around the division was calculated as a function of distance to the division and normalized to the speed of the rest of the layer. The same procedure was applied to up to 20 images, corresponding to 60 min, before and after the detection of the mitosis event. This period was chosen to assess the effect of pre-mitotic contraction and post-mitotic expansion. To further assess the cell density in each field of view, the initial cell number at t = 0 h was manually counted in a sub-field of 144 × 144 μm² and extrapolated to the whole field of view. To calculate the cell density *ρ* over the whole time, for each field of view the number of proliferations was added to the initial cell density. Combined with the layer migration speed *v* this approach allowed to calculate the scaling coefficient *α* between both quantities as follows:

$$\:v\:\sim \:\rho \:^{{\alpha \:}}$$

Obtaining the number of proliferation events and the initial cell density allows the calculation of doubling times for each cell line. Therefore, the current cell number *N(t)* at time *t* in a field of view and the cell number at Δt = 12 h later were used to estimate the instantaneous doubling time t_d_ as follows:

$$\:t_{d} = \frac{{\Delta \:t{\mathrm{*}}\:N\left( t \right)}}{{N\left( {t + \Delta \:t} \right) - N\left( t \right)}}$$

### Estimating the contribution of proliferation events to monolayer speed

The contribution of division events to the overall layer speed was estimated by the temporal and spatial contribution of pre-mitotic contraction and post-mitotic expansion obtained before. This allows to distinguish between all points in the image that are affected by proliferation events up to 1 h before and after the current time point and those that are currently not affected, classifying each data point as either “unaffected by proliferation events” or as “affected by proliferation events”. The “unaffected regions” were used to define the baseline speed, and the ratio of the speeds of both regions was considered as the relative contribution of proliferation events to the overall layer speed. Notably, the first and last hour of measurement, and the edges of images were discarded, as no data were available about proliferation events outside of the field of view or before and after the measurement.

### Analyzing self-organization in monolayers

To determine local orientation of cells in a layer the largest eigenvector of the structure tensor *J* of the image *I* was used. The structure tensor *J* was defined as^42^:

$$J_{{pq}} \left( {\vec{x}} \right) = \:\int \: w(\vec{x} - \vec{x}\prime \:)\left( {\frac{{\partial \:I\left( {\vec{x}\prime \:} \right)}}{{\partial \:\vec{x}\prime \:_{p} }}\:\frac{{\partial \:I\left( {\vec{x}\prime \:} \right)}}{{\partial \:\vec{x}\prime \:_{q} }}} \right)\:d^{2} \vec{x}\prime \:\:\:\; with \; p,q\: \in \:\:[x,y]\:$$

and the Gaussian window function *w*. From the cellular orientation, the (local) nematic order parameter *S* was calculated as:

$$\:S = \: < 2*{\mathrm{cos}}^{2} \theta \: - 1 >$$

With *θ* being the angle between the cellular orientation and the mean orientation of all cells, either in the whole field of view or a defined sub-window. To evaluate the spatial dependence of the order parameter, a power law was fitted as $\:S\left(l\right)\sim\:{l}^{a}$, with the decay exponent *a* and the length scale *l*^35^. For long range order *a* approaches 0 and *S* remains constant. For 0 > a>-1 the order is considered to be of a quasi-long range type, with the extreme case a = -1 corresponding to a random order of orientations. For short-range order, an exponential decay is expected, but for the data given here, a power law describes the data best.

As nematics contain topological defects, ± 1/2 defects were identified using the Matlab toolbox “defector find”^43^ on the previously calculated cellular orientation fields. This toolbox calculates the winding number around potential topological defects in order to classify them as ± 1/2 defects. Notably, this allows assessing the evolution of the number of topological defects as a function of time and the movement of topological defects.

To evaluate if the ordering might be of a polar type, a polar order parameter *V* was introduced^32^:

$$\:\:V = \:\frac{1}{N}\:\sqrt {\left( {\sum {\:_{{i = 1}}^{N} } {\mathrm{sin}}\vartheta \:_{i} } \right)^{2} + \:\left( {\sum {\:_{{i = 1}}^{N} } {\mathrm{cos}}\vartheta \:_{i} } \right)^{2} }$$

Here, *N* corresponds to the number of angles *ϑ* in the whole field of view or a defined sub-window. *ϑ* is the angle between cellular orientation and velocity at the same spatial position. Similar to the nematic order parameter, the decay of the polar order parameter as a function of coarse graining was assessed. Here, an exponential decay function fitted the data best:

$$\:V\left( l \right) = m*e^{{ - \frac{l}{{l_{0} }}}} + V_{0}$$

With the characteristic decay length *l*_*0*_ and the constant offset *V*_*0*_.

### Cell exclusion assay

For the cell exclusion assay 50,000 cells were seeded per well in a confined ring structure in 12-well plates. Twenty-four hours after cell seeding, the cells reached confluence. Thereafter, the ring structure was removed, and cells were transferred to the microscope (Leica DMi8, Leica, Wetzlar, Germany, 5% CO_2_ (v/v), 37 °C) for monitoring cell expansion. Five regions of interest were imaged per well. Cell expansion was monitored for 60 h with images taken every 5 min.

To analyze cell expansion a U-Net was trained to segment the cell layer from the background. Local velocities and other quantities were calculated as described under “Collective Cell Migration”, with the restriction of the analyzed area to the cell covered area.

For assessment of cell density dependent effects, another U-Net was trained to identify cell-cell boundaries in phase contrast images and thus assess cell sizes inside the layer. For training 52 manually segmented sample images, containing 15,015 cells, were used. For training and later analysis, the original images of size 1280 × 960 pixels were split into batches of 256 × 256 pixels. From each training image, 64 random patches of the batch size were chosen and randomly augmented as follows: random shearing in x- and y- direction from − 15° to 15°, random rescaling by factors of 0.7 up to 1.3, and random rotations from 0 to 360°. The U-Net was trained for 50 epochs, with a learning rate drop factor of 0.8 and an initial learning rate of 0.5 *10^− 4^.

The confusion matrix, accuracy and F1-score for the pixel wise segmentation of the test data set were calculated to test the trained U-Net. In addition, an estimate of the goodness of cellular identification was performed. Therefore, the raw segmentation was post-processed as follows: disconnected objects smaller than 50 pixels were removed, followed by morphological closing and skeletonizing of the binary image. For skeletonizing a minimal branch length of 150 pixels was chosen. For matching, the centers of the segmented cells were compared to the cell centers obtained from the ground-truth measurements. If the distance between the centers of an automatically and manually segmented cell was less than 4.8 μm (5 pixels), the segmented object was declared as true positive. True positives were considered as correctly identified cells, false positives as segmented cells with no sufficiently close (4.8 μm) cell in the ground truth image and false negatives as cells in the ground truth image with no sufficiently close (4.8 μm) cell in the segmented image.

To further improve the detection of cells, too small (less than 120 μm²) and too large (more than mean size + 2 * median absolute deviation of size) segmented objects were discarded. Furthermore, segmented objects have been assigned to tracks via the Munkres global nearest neighbor algorithm and segmented objects that have not been assigned in at least 4 of the 5 following images were discarded too, to reduce the number of misdetections. The following energy function was used as input for the Munkres algorithm for the assignment of detection *d* to track *t*:

$$\:Cost\:\left( {d,t} \right) = \left( {1 + n} \right)*\Delta \:x\left( {d,t} \right)*(1 + |\Delta \:A\left( {d,t} \right)|)$$

Here, *n* corresponds to the number of frames the detection *d* was not assigned to any track *t*. *Δx(d*,* t)* was the normalized distance of the detection *d* to the last detection of track *t*. *ΔA(d*,* t)* corresponds to the normalized difference in cell size between the currently detected cell *d* and the last detection of track *t*. If the assignment cost for a detection was too high, a new track was created.

To calculate the gradient of the cell density in a field of view, the cell density in dependence of the distance to the cell cluster boundary was calculated and linearly fitted. The slope of the fit was used as cell density gradient estimate.

To assess the relation between the expansion rate of the monolayer and the cell density gradients, piece-wise linear fitting was employed. To identify segments, the method introduced by R. S. Bogarts was used^44^.

### Statistics

Statistics were performed using the two-tailed ANOVA with Tukey post-hoc test or the two-sided sign test. Significance was defined for *p* < 0.05. All error-bars and shaded areas depict the standard error of the mean. Experiments were repeated at least three independent times. For live-cell imaging experiments assessing collective motion, 4–8 fields of views were measured per experiment and condition. Confidence intervals for fit parameters were obtained using the MatLab function confint.

## Results

### Glioblastoma show cell type dependent response to increasing cell density

**Fig. 1:**
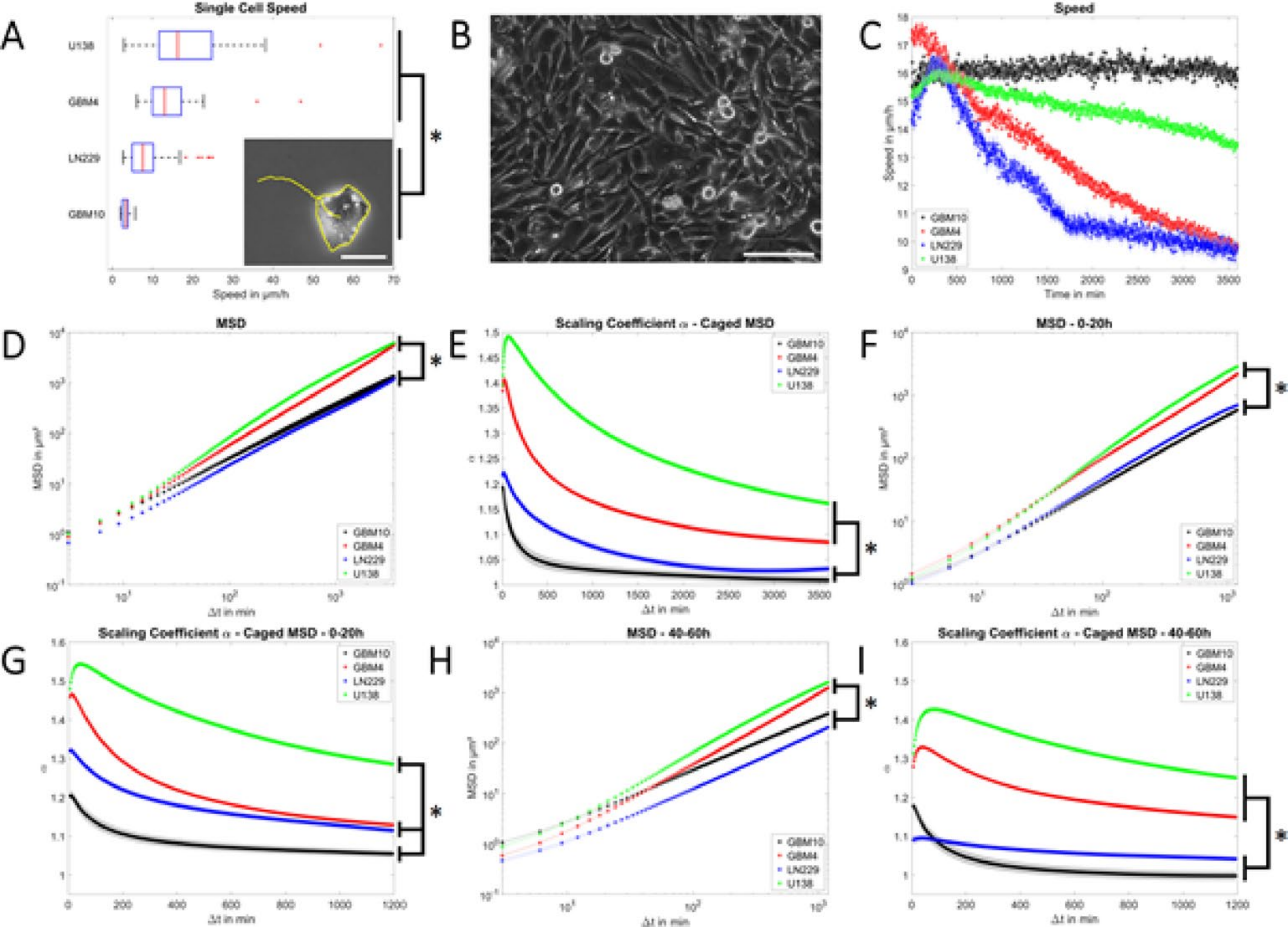
Migratory capacity of GB cells. (**A**) Graph of the single cell migration speed, with the inlet showing the path and edge detection of a LN229 cell. Scale bar corresponds to 25 μm. (**B**) Image of a dense monolayer of U138 cells at 0 h, illustrating the experimental starting point. Scale bar corresponds to 125 μm. (**C**) Depiction of the mean layer speed as a function of time for all 4 GB cell lines. (**D**) and (**E**) Analysis of the mean squared displacement and the scaling coefficient of all cell lines for the whole 60 h period. (**F**) to (**I**) Illustrations of the same parameters as in (**D**) and (**E**) but for the first 20 h for (**F**) and (**G**), or the time from 40 h to 60 h in (**H**) and (**I**). Please note that the movement pattern of U138 and GBM4 cells is less affected than that of LN229 and GBM10 by increasing time and thus cell density. Error bars and shaded areas depict the standard error of the mean. Box plots show the median (red line), 25 and 75 percentile (box), non-outlier range (whiskers) and outliers (red dots). Stars depict significant differences between the last time point of groups with *p* < 0.05 calculated using a two-tailed ANOVA with the Tukey post-hoc test. Sample sizes: (**A**) n_U138_ = 44; n_GBM4_ = 47; n_LN229_ = 83; n_GBM10_ = 43; (**C**) to (**I**): n_U138_ = 184; n_GBM4_ = 139; n_LN229_ = 79; n_GBM10_ = 60;

To establish the individual migratory potential of all four GB cell lines, we first analyzed the single cell motility. GBM4 and U138 moved significantly faster and tended to move more directed than LN229 and GBM10 (Fig. 1A, Fig. S1). The movement speed in a dense monolayer (Fig. 1B) did not follow the same pattern, as U138 and GBM10 moved fastest (Fig. 1C). Nonetheless, GBM4 and U138 moved more effectively than LN229 and GBM10 as demonstrated by the higher MSD and scaling coefficient (Fig. 1D-I). The overall speed of GB cells seemed to mostly decrease over time (Fig. 1C). When analyzing the scaling coefficient of the MSD at the beginning and end of the measurement period, corresponding to low and high cell densities, a cell type dependence was found (Fig. 1F-I). LN229 and GBM10 showed lower scaling coefficients over time, approaching a diffusive type of motion for high cell densities, while U138 and GBM4 cells were less affected and continued to show a super-diffusive type of motion (Fig. 1F-I).

Next, different aspects of a changing cell density were evaluated to elucidate potential causes for the cell type dependent response. Three different types of changes in cell density were assessed: (1) Local changes of cell density caused by individual proliferation events, (2) response of cell layers when subjected to cell density gradients and (3) the effect of a global increase in cell density.

### Proliferation events cause a local acceleration in glioblastoma

As proliferation is a ubiquitous process present throughout all our experiments, we first evaluated whether isolated proliferation events affect migration. To test this hypothesis, a dense, homogenous monolayer was investigated, which is largely independent of cell density gradients. A data set consisting of 462 regions of interest was examined, including approximately 134,000 cell division events (Table S1).

**Fig. 2:**
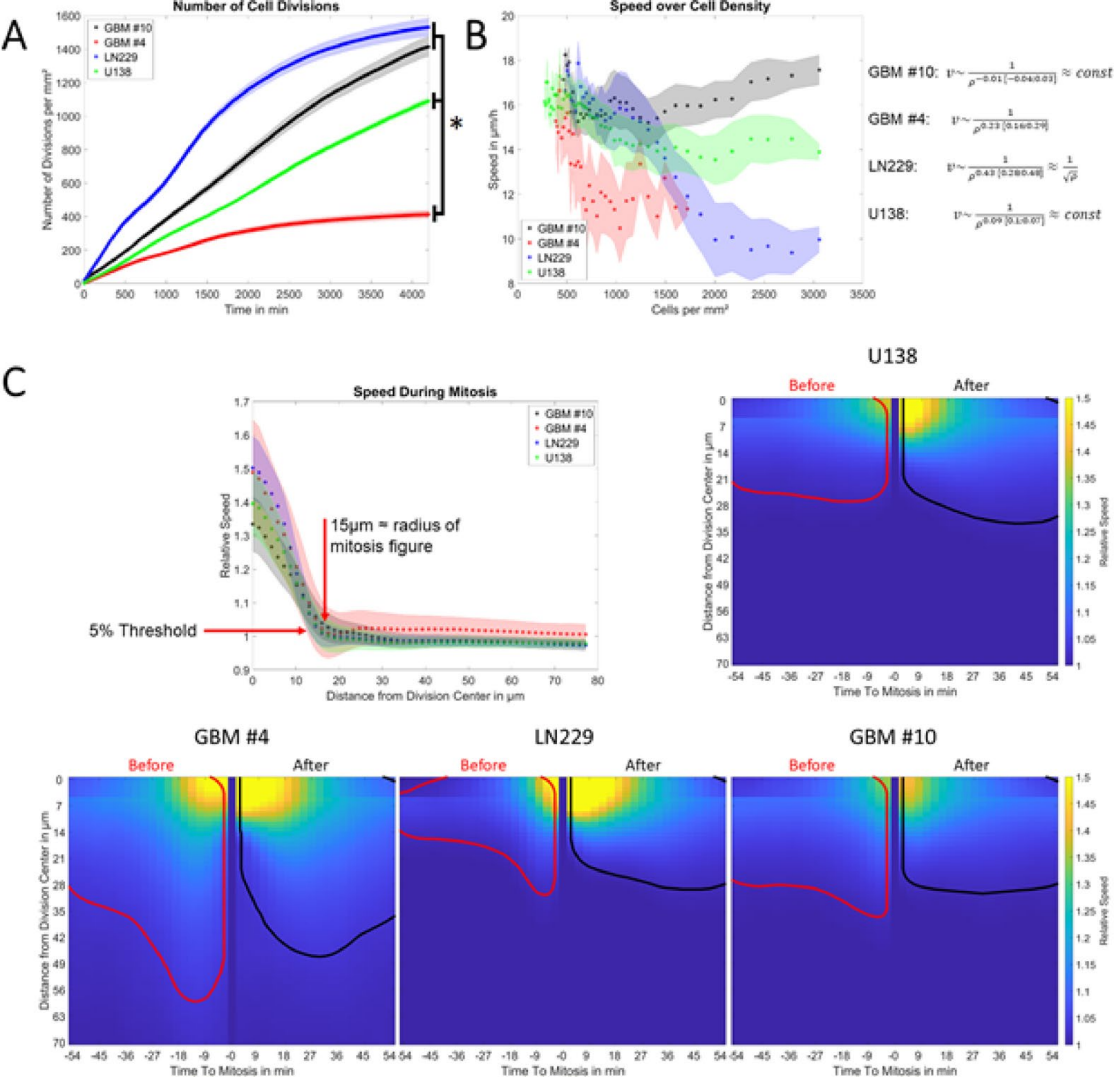
Analysis of proliferation and its effect on migration speed. (**A**) Graph of the number of proliferation events as a function of time. Please note that the less-migratory LN229 and GBM10 cells have a higher proliferation rate. (**B**) Plot of the layer speed as the function of cell density, showing cell type dependent behavior and validating previously found scaling coefficients. (**C**) Quantification of the effect of proliferation on velocity fields. The top left graph demonstrates that mitotically rounded cells do not affect the motility of cells around them significantly. The remaining graphs illustrate the effect of pre-mitotic cell contraction and post-division spreading. The encircled regions depict the spatial and temporal regime were pre-mitotic contraction (red) or post-mitotic spreading (black) led to a measurable increase in velocity (> 5%) in the surrounding cells. Error bars and shaded areas depict the standard error of the mean. Stars depict significant differences between the last time point of groups with *p* < 0.05 calculated using a two-tailed ANOVA with the Tukey post-hoc test. Sample sizes: (**A**) to (**B**): n_U138_ = 184; n_GBM4_ = 139; n_LN229_ = 79; n_GBM10_ = 60; C) n_U138_ = 58,131; n_GBM4_ = 16,645; n_LN229_ = 34,993; n_GBM10_ = 24,612;

To study the effect of proliferation events, we first validated our approach for detecting division events. Therefore, the cell density at t = 0 h was manually determined for each region of interest (Fig. S2), and estimated for all subsequent measurement times by automatic detection of cell division events. This allowed calculating and comparing the scaling coefficients between cell density and layer speed for U138 and LN229, with previously published data^12^(Fig. 2A, B). Scaling coefficients differed by only ± 0.01, which is in good agreement. In addition to previously performed tests^31^, a reliable capability to track division events could be demonstrated. GBM10 and LN229 showed significantly more proliferation events than GBM4 and U138 (Fig. 2A). Calculating the instantaneous doubling times, a time dependent increase was observed, reaching values of 40 days or more for LN229 and GBM4 cells, implying a growth arrest like state (Fig. 2A, Fig. S3). To further validate our approach, the mean doubling time for the first 15 h was taken as an estimate of the doubling time for each cell line, giving values of 2.0 d (± 0.2 d) for GBM10, 1.8 d (± 0.2 d) for LN229 and 2.5 d (± 0.2 d) for U138. No values for GBM4 were calculated, as a relatively constant regime was missing. Notably, the values for the commercially available cell lines corresponded roughly to those previously reported for LN229 (0.8 d to 1.1 d) and U138 (2.0 d to > 3.3 d) obtained under non-confluent conditions^45–47^.

**Fig. 3:**
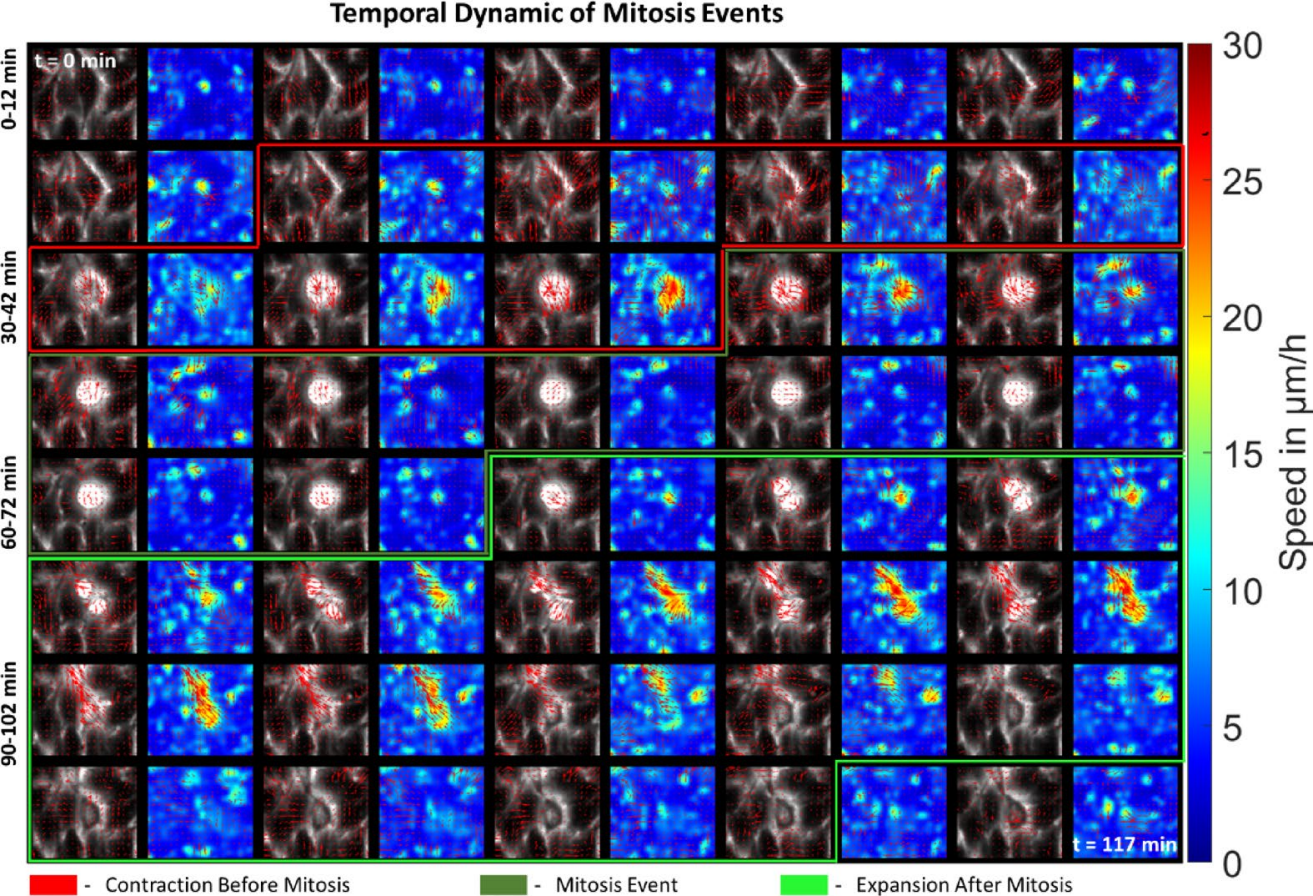
Temporal dynamic of a mitosis event. This graph illustrates the temporal dynamics of a mitosis event over 2 h, combined with the accompanying changes in the surrounding velocity field. It can be found that the process leading to mitotic rounding affects the speed of the surrounding cells, as does the daughter cell expansion after mitosis.

A visual examination of the velocity field of cells around proliferation events was performed to assess qualitatively whether proliferation was associated with changes in migration (Fig. 3). Three distinct phases were identified, namely a phase of pre-mitotic contraction, a mitotically rounded phase, and a post-mitotic expansion phase. During pre-mitotic contraction, the velocity of the surrounding cells increased (Fig. 3, red), but when cells were fully mitotically rounded the velocity slowed down (Fig. 3, dark green). During daughter cell expansion, an increase in speed was observed (Fig. 3, light green) before an eventual normalization. These qualitative observations motivated the distinction of the three different phases for quantitative analysis.

For quantification of the proliferation-induced changes in the velocity field, the speed was analyzed as a function of the distance to the dividing cell during the time a cell was identified as mitotically rounded, for all regions of interests and all cell densities. The speed around the mitosis event was found to be higher at the core of the event, but dropped quickly, reaching a value that is mostly indistinguishable from the background speed (≤ 5% increase) for distances of 15 μm or higher, corresponding approximately to the radius of the mitosis figure (Fig. 2C). Thus, during mitosis the overall layer migration speed was not altered significantly. Based on these measurements the cut-off point of 5% appeared to be reasonable for determining a region that is affected by proliferation events.

Afterwards the time up to 1 h before the start and up to 1 h after the end of mitosis was analyzed and the distance dependent speed calculated. Pre-mitotic contraction and post-mitotic expansion led to an increase in the speed of the surrounding layer (Fig. 2C, heat maps). The spatio-temporal pattern of both parts was different. The effect of pre-mitotic contraction tended to be smaller the further in time the mitosis event was and reached its peak, in effective range and overall magnitude, approximately 10 min prior to mitosis. The effect of the post-mitotic expansion built up its effective range over time, reaching its maximal range approximately 30 to 40 min after mitosis (Fig. 2C, heat maps).

To elucidate whether the effects of cell divisions were dependent on the cell density, the maximal effect range – defined as the highest distance with an increase in speed ≥ 5% relative to the background – was analyzed as a function of cell density. For all cell lines and pre- and post-mitotic phases, increasing cell density reduced the range in µm affected by a division event. This effect was counterbalanced by the increasing cell density, resulting in an effective increase in the number of cells affected by a proliferation event. Thus, cells three or more cell radii away from a division event were affected (Fig. S4).

Next, we calculated the average area affected by proliferation events. Approximately 20–30% of the monolayer were on average influenced by cell division events for each cell line (Fig. S5). These numbers were used – together with the data from Fig. 2C – to estimate the proportion division events had on the total speed. However, the estimated proportion was ≈ 4–5% for all cell lines, suggesting that individual proliferation events were not the main driver of cell migration in GB in our system and cannot explain the phenotypic differences between the cell lines. Therefore, the effect of cell density gradients on migration was addressed as the next step.

### Cell density gradients can be a driver of glioblastoma migration but in a cell type dependent manner

**Fig. 4:**
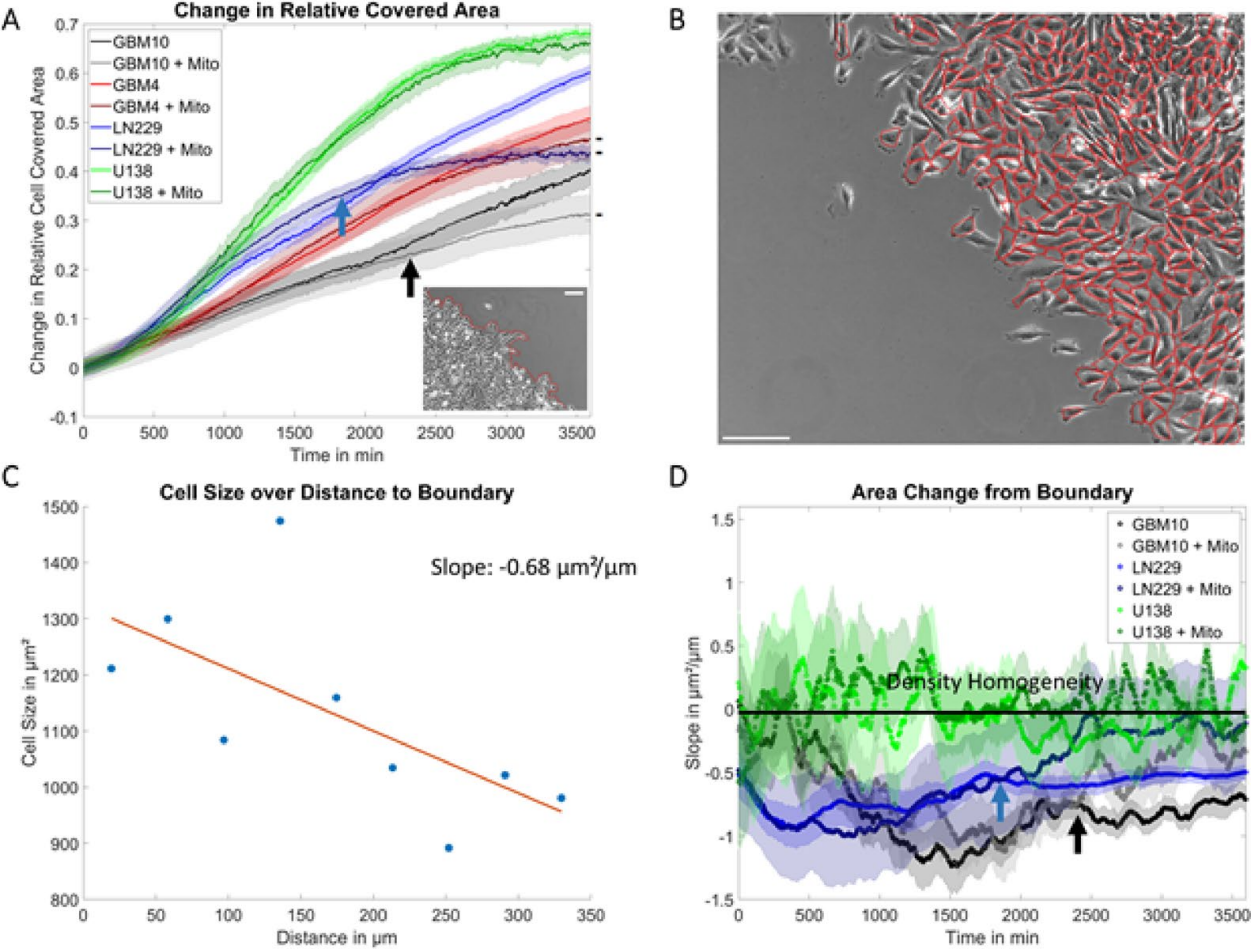
Analysis of GB cell expansion in presence and absence of proliferation. (**A**) Shows the expansion rate of GB cells under control conditions and when treated with mitomycin C to block proliferation. For U138 saturation occurs as the whole field of view is eventually covered by the cells. Blue and Black arrows show the same time point as in (**D**), roughly corresponding to the points were the expansion rate of mitomycin C treated LN229 and GBM10 cells diverged from the controls. The inlet shows a typical sample image of the cell exclusion assay for LN229 and the associated detected cell front. Scale bar corresponds to 100 μm. (**B**) Image of the final cell segmentation for LN229 cells, showing good segmentation results. Scale bar corresponds to 75 μm. (**C**) Plot of the cell size as a function of distance of the cell to the cell front for LN229 cells, including a linear fit and its slope, corresponding to the cell density gradient. Please note the decline in cell size with increasing distance from the front. (**D**) Graph of the cell density gradient as a function of time for mitomycin C treated and untreated cells. The black line shows the reference for the absence of any cell density gradient. Arrows mark the time points at which the cell density gradients between the control and mitomycin C treated LN229 and GBM10 cells start to diverge. Error bars and shaded areas depict the standard error of the mean. “-“ depicts a significant reduction relative to the untreated control group for the last time point with p < 0.05 calculated using a two-sided sign test. Sample Sizes: (**A**), (**D**) n_U138 CTL_ = 24; n_U138 Mito_ = 11; n_GBM4 CTL_ = 39; n_GBM4 Mito_ = 10; n_LN229 CTL_ = 29; n_LN229 Mito_ = 15; n_GBM10 CTL_ = 13; n_GBM10 Mito_ = 13.

To generate a cell density gradient, cells were seeded in a confined circular space. After removal of the confinement, the expansion of the layer was measured. Analysis of the cellular speed as a function of distance to the cell front showed that the layer expansion was initiated at the front and gradually spreading deeper into the cell layer (Fig. S6). In the presence of proliferation, the previous hierarchy in terms of migration was no longer preserved, showing that U138 and LN229 covered a larger area than GBM4 and GBM10. Among the here investigated cell lines LN229 and GBM10 had the highest proliferation rate, combined with lower migratory capacity in terms of MSD, scaling coefficient and single cell speed indicating that cell density gradients could drive layer expansion. To test this hypothesis, proliferation was inhibited by addition of a non-lethal dose mitomycin C, as verified by analysis of proliferation events (Fig. S7). Expansion of mitomycin C treated cells was similar to the control in the first ≈ 2000–2200 min. Then, for all cell lines but U138, the treated cells were expanding slower than controls, and for LN229 and GBM10 an arrest of expansion was observed (Fig. 4A). In the absence of proliferation, U138 and GBM4 were eventually expanding faster than LN229 and GBM10. To demonstrate a potential dependence of migration on the cell density gradient, a U-Net was trained to segment individual cells. Testing the neuronal network in an independent image batch, a pixel-wise accuracy of 84.7% and an F1 score of 70% was found (Table S2, Fig. S8). At the level of individual cells the obtained F1 score was 77% (Table S2), demonstrating the feasibility of the trained network for segmentation. Together with sized based filtering and track assignment, the detection gave good results for all but one cell line (Fig. 4B). Only for GBM4, no good segmentation results were obtained, as no manual ground truth could be created, as cell edges were often not clearly discernable (Vid. S1).

Using this segmentation approach for each image, the cell size was determined as a function of distance to the cell front, and its slope calculated as a measure for the cell density gradient (Fig. 4C). For LN229 and GBM#10 in control conditions, the cell density gradient decreased after removing the confinement, reaching a minimal value and afterwards increased to a constant level of ≈-0.5 μm²/µm or ≈-0.7 μm²/µm, respectively (Fig. 4D). Mitomycin C treated cells behaved similarly, until ≈ 1850 min or ≈ 2300 min, when the cell density gradient started to increase above the equilibrium of the controls, to a state of homogeneous cell density. The time the cell density diverged between control and mitomycin C treated cells corresponded to the time the cluster expansion diverged as well. Upon reaching equilibrium cell size, cluster expansion almost ceased, suggesting that expansion of GBM10 and LN229 was driven by cell density gradients (Fig. 4A and D). This was supported by the increase in average size for both cell lines after mitomycin C treatment. The scatter plots of the cell density gradient over the expansion rate showed a negative correlation between cell density gradient and layer expansion rate for LN229 and GBM10 (Fig. S9, Table S3). Notably, the dependence of the expansion rate on cell density was cell type dependent. For LN229 an initial negative correlation of expansion and cell density gradient was observed for cell density gradients up to -0.38 μm²/µm, followed by a constant regime of approximately zero expansion rate (Fig. S9C, Table S3). For GBM10 an initial constant regime was observed for cell density gradients up to -0.65 μm²/µm, followed by a negative correlation of expansion rate and cell density gradient (Fig. S9D, table S3). For U138 cells, no consistent deviation of the cell density gradient from zero was observed, independent of the presence or absence of proliferation. Thus, the expansion of U138 was not determined by cell density gradients, as supported by the piece-wise linear fitting procedure, showing no breaking points and only one regime that was indistinguishable from a constant model (Fig. S9B, Table S3). Notably, no large qualitative differences were found in cluster cohesiveness between groups, capable of explaining those differences. For all cell lines, a high cohesiveness of the front was observed, with only few cells escaping the layer (Vid. S1-S5).

### Glioblastoma show self-organization in liquid crystal-like structures

**Fig. 5:**
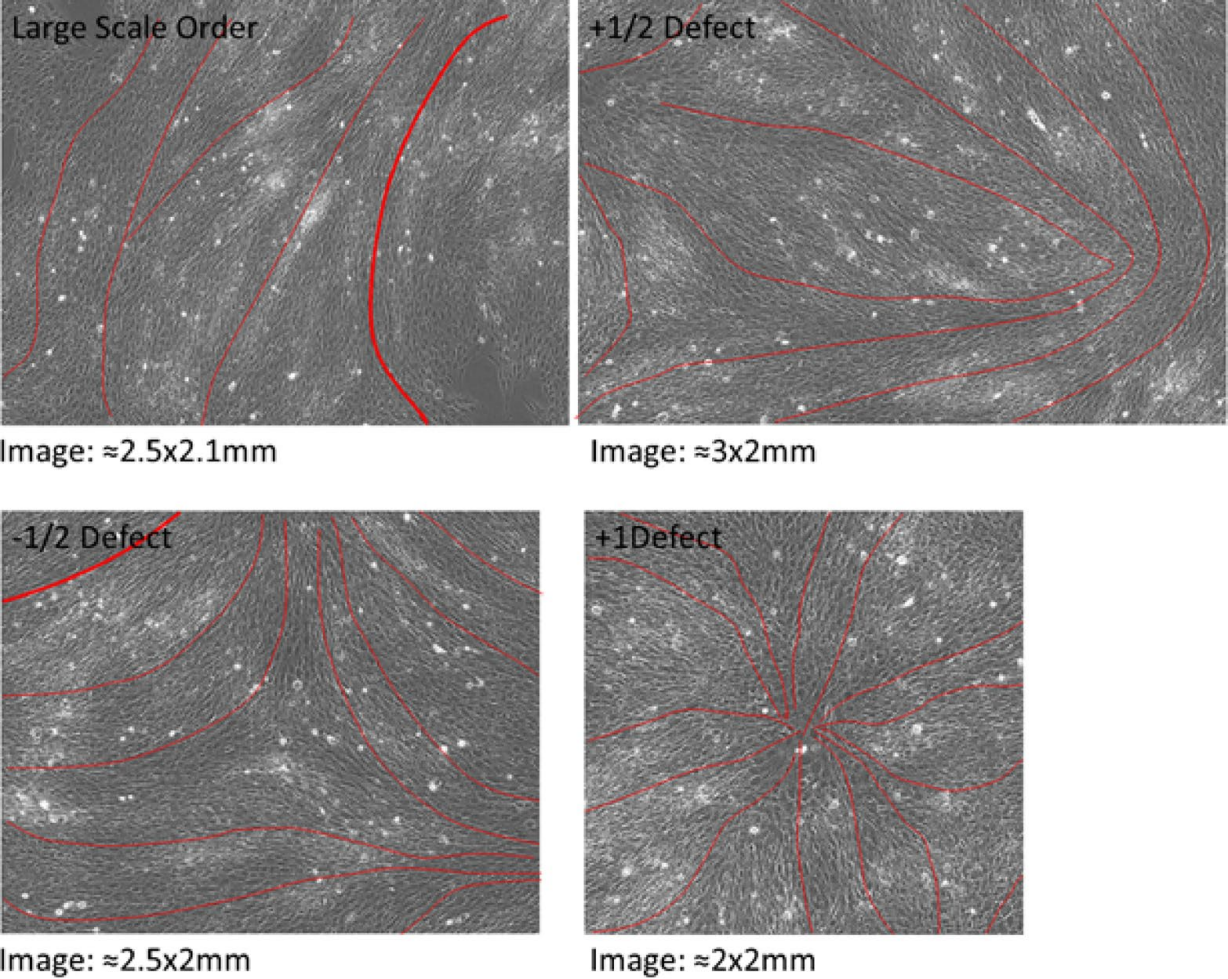
Topological defects found in GB cultures. The top left image shows GBM4 cells in a highly organized state, mostly aligned in one direction. The top right image depicts GBM4 cells organized in the form of a + 1/2 or comet like defect. The bottom left image illustrates the formation of -1/2 or trefoil defects in GBM4 cells, and the bottom right image is that of a rarely observed + 1 or aster defect.

**Fig. 6:**
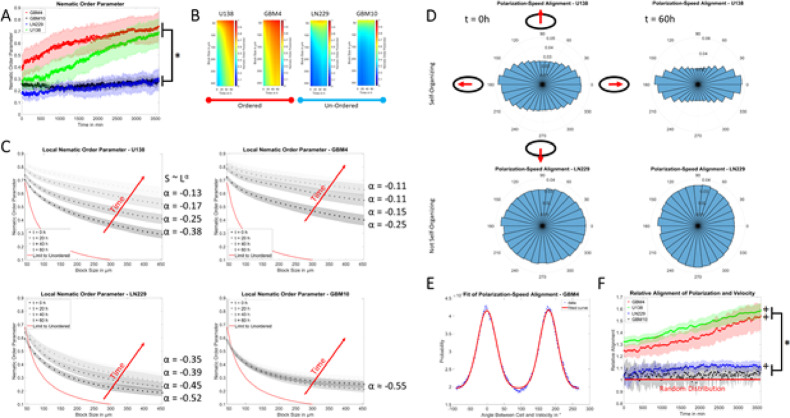
Nematic order in GB cells. (**A**) Plot of the temporal evolution of the nematic order parameter for the whole field of view. (**B**) Heatmaps of the temporal and spatial evolution of the order parameter for all four GB lines. (**C**) Graphs of the nematic order parameter as a function of the used spatial scale and defined time points. Red lines show the expectation value for a random distribution of orientations and the red arrow depicts the direction of increasing time. To the right of the graphs the exponents of the power law fits for each chosen time are shown. (**D**) Polar plots of the alignment of cellular orientations with the velocity vectors at the beginning (left) and after 60 h (right) for U138 (top) and LN229 (bottom) cells. (**E**) Sample distribution of the angle between cell orientation and velocity for one field of view of GBM4 cells, together with the respective fit in red. (**F**) Relative alignment of cellular orientation and velocity field, in terms of the number of cells moving in ± 22.5° of their orientation, over time. The red line shows the value expected for a random alignment. Error bars and shaded areas depict the standard deviation. Stars depict significant differences between the last time point of groups with *p* < 0.05 calculated using a two-tailed ANOVA with the Tukey post-hoc test. “+“-signs depict values of the last time point for groups significantly larger than one, with p < 0.05 calculated using a two-sided sign test. Sample sizes:  (**A**) to (**C**), (**F**): n_U138_ = 184; n_GBM4_ = 139; n_LN229_ = 79; n_GBM10_ = 60;

To analyze the effect of increasing cell density, independent of cell density gradients, the confluent monolayer system of homogeneous cell density was used. As observed previously^31^ and here (Fig. S10, Vid. S1, Vid. S2), GBM4 and U138 showed signs of self-organization for high cell densities that were reminiscent of oncostreams^34^, not visible in the other two cell lines. Thus, those structures might be involved in the sustained migration for high cell densities. To assess the type of structures formed, monolayers were analyzed manually to identify breakage points in the underlying structures, termed topological defects. Three types of defects were identified: +1/2 defects (comet), -1/2 defects (trefoil) and + 1 defects (aster, Fig. 5). From all defects found throughout this study, only two were + 1-type defects but the vast majority were of the ± 1/2-type, and therefore the underlying organization was of a nematic type. Consequently, the nematic order parameter was used to assess the evolution of structure formation over time. In GBM4 and U138 the nematic order parameter increased over time and thus cell density, regarding the whole field of view (≈ 0.29 mm², Vid. S6, Vid. S7). GBM10 and LN229 cell lines showed only little nematic ordering that was slightly increasing over time (Fig. 6A, Vid. S8, Vid. S9). Analyzing the nematic order for different spatial dimensions ranging from windows with edge length of 25 μm to 325 μm a decreasing nematic order was found for increasing window size. For LN229 there was an increase in the local nematic order over time, spanning cell populations of the size of up to ≈ 0.04 mm², implying the evolution of a locally ordered state (Fig. 6B). For GBM10 the nematic order did not change strongly over time, independent of window size (Fig. 6B). To evaluate the range of order, the decay of the order parameter was calculated in dependence of the analyzed window-size. This decay function was best fitted by a power-law, with values of the exponents approaching ≈-0.1 for GBM4 and U138, implying a high order on a large scale (Fig. 6C). For LN229 cells, the scaling coefficient increased from − 0.52 to -0.35, implying a gradually stronger ordering (Fig. 6C). In contrast, in GBM10 the scaling coefficient stayed roughly constant around ≈-0.55 (Fig. 6C). The results are in line with the qualitative assessment of the emergence of order from before. To validate the increasing organization of GB cells over time, the number of ± 1/2 defects was evaluated as a function of time. A strong decrease of defects was detected in U138 and GBM4, a less pronounced decrease in LN229 and a constant number in GBM10, being in line with the expectations based on the nematic order parameter (Fig. S11-14E). The number of + 1/2 and − 1/2 defects was, as expected, almost identical for all cell types validating these results further. To assess whether the defects themselves were associated with cell motion, their MSD and movement path relative to the defect orientation were analyzed for defects persisting at least 3 h. Defects showed a sub-diffusive movement pattern, with no favored direction relative to their orientation (Fig. S11-14 A-D).

### Liquid crystal-like self-organization promotes migration in high cell density limit

The alignment of velocity vectors and cellular orientation were studied to test whether the nematic order is related to the directionality of migration. For LN229 and GBM10 the alignment of cells and velocity resembled a random distribution. For GBM4 and U138 both vectors tended to align in parallel or anti-parallel, with increasing alignment over time and cell density, implying the formation of anti-parallel moving streams (Fig. 6D, E). For quantification, the probability density function was fitted as the sum of two Gaussians and a constant offset (Fig. 6E). To quantify the proportion of cells moving in the direction of cellular alignment, the proportion of cells located at the peak centers ± 22.5° was calculated and normalized to the expectation value for a uniform distribution. For GBM4 and U138 the velocity-cellular-orientation alignment was significantly higher than expected by chance. The velocity-cellular-orientation alignment increased over time and thus cell density. Whereas this value was close to the value expected by chance for GBM10, a slight increase over time was observed for LN229 cells (Fig. 6F). As these measurements suggested the formation of anti-parallel moving streams of cells, the emergence of polar order was tested. Therefore, the polar order parameter for the velocity field was calculated. Only small differences were found between cell lines, and the polar order was smaller than the nematic order, staying almost constant over time and thus cell density (Fig. S15). The decay of the velocity order parameter was best fitted by an exponential decay, implying a short-range order. The decay coefficients stayed approximately constant for all cell types over time with values between ≈ 90 and ≈ 140 μm, depending on the cell line. Therefore, the order in GB is most likely of a nematic and not of a polar type.

**Fig. 7:**
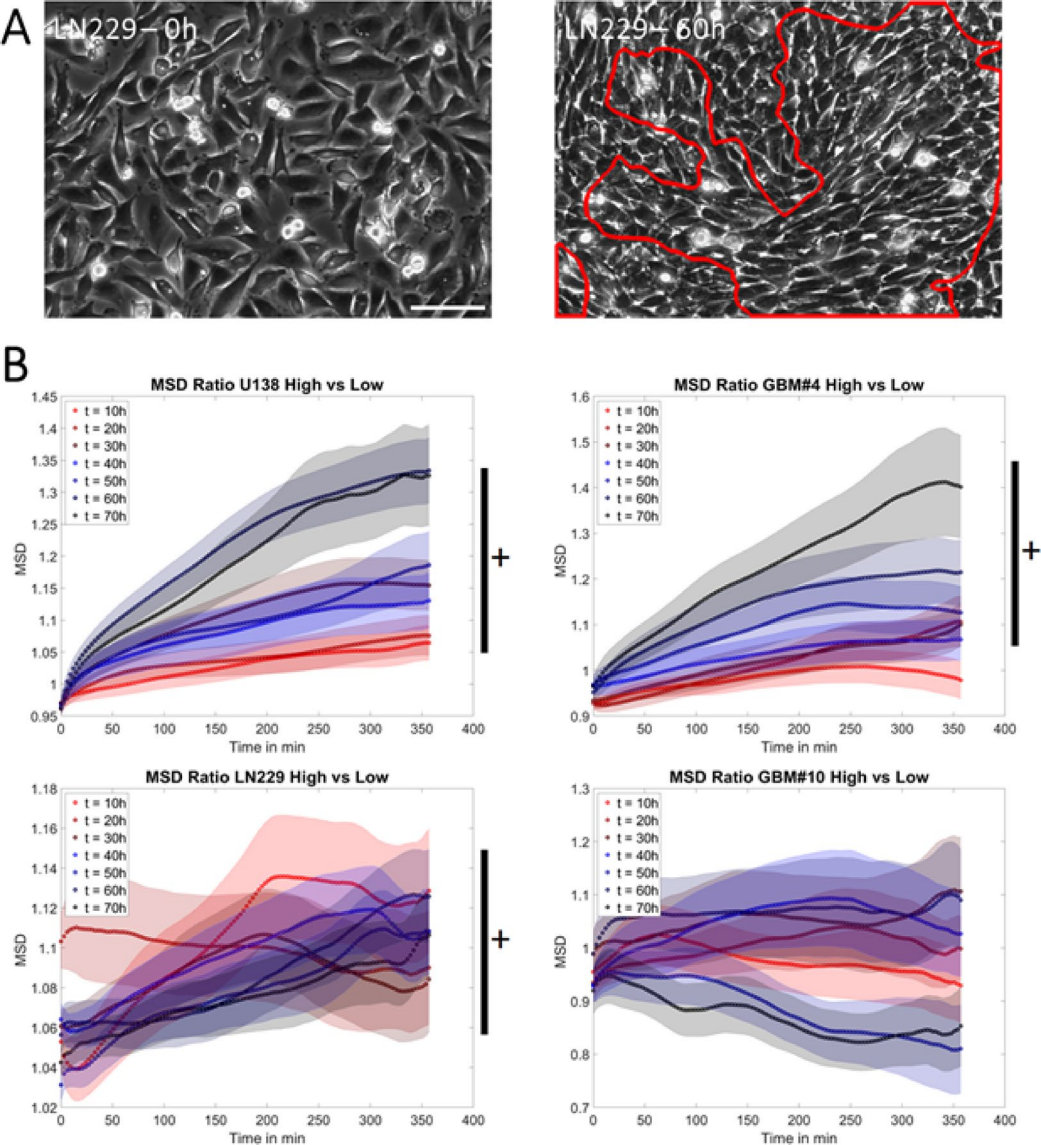
Comparison of the migration of ordered and unordered regions in one field of view. (**A**) Example image of the emergence of local nematic order in LN229 cells. The left image shows the cell layer at 0 h, while in the right image taken after 60 h a locally ordered region, encircled in red, has developed. Scale bar corresponds to 100 μm. (**B**) Ratios of the mean squared displacement for regions with high nematic order over those with low nematic order for U138 (top left), GBM4 (top right), LN229 (bottom left) and GBM10 (bottom right). Error bars and shaded areas depict the standard error of the mean. “+“-signs depict values of the last time point for groups significantly larger than one, with p < 0.05 calculated using a two-sided sign test. Sample sizes for t = 0,10,20,30,40,50,60 h: (**B**): n_U138_ = 180, 167, 161, 145, 135, 101; n_GBM4_ = 139, 131, 122, 115, 106, 88; n_LN229_ = 79, 79, 78, 78, 77, 73; n_GBM10_ = 32, 27, 28, 29, 28, 21;

These measurements suggest that nematic order might be related to a consistent movement. If this is valid, it is expected that inside a monolayer of one cell type, groups of cells showing high nematic order (S > 0.7) migrate more effective than groups of lower order (S < 0.3). To test this hypothesis, all imaged regions were examined whether they simultaneously contained regions of low and high nematic order (Fig. 7A). The regional threshold was set to 1820 μm² to contain multiple cells. The movement of those clusters was tracked for the following 6 h. The highly ordered cells in U138 and GBM4 were slower (Fig. S16), but moved more effective, as shown by higher MSD. The effectiveness of motion was increasing over time and thus cell density (Fig. 7B). Similarly, ordered clusters of LN229 moved more effective, but no significant changes in speed were observed (Fig. 7B, Fig S13). For GBM10 no such effect was observed (Fig. 7B), but due to the overall low ordering of this cell line only few regions of interests contained areas of high and low ordered cell groups.

## Discussion

In this manuscript, the effect of various changes in cell density on the migratory capacity of GB cells was analyzed. Two different phenotypes were identified: one being mostly independent of cell density and the other showing an effective slowdown of cell body translocation with increasing cell density. Our analysis suggested that the organization of cells in a liquid crystal-like structure is associated with persistent cellular mobility even under conditions of high cell density.

Under confinement and with increasing cell density, studies suggested that cellular movement slows down, leading to a migratory arrest^6–13,16^. This happened with an optimal motility at intermediate cell densities^16^. Yet, other studies showed opposing results with cells staying motile for high cell densities^12,48^.

For cells to be able to escape the confinement for high cell densities, sufficient forces need to be generated. One cause are proliferation events^30^. Thereby, cells were considered to generate forces in different phases. During mitotic rounding a net inward stress^20,21,29^, caused by acto-myosin contractility and increased hydrostatic pressure, is generated^24,49–53^. During the subsequent daughter cell expansion, cells generate outward stress^20,29^. For both phases, we observed an increase in the local velocity around the dividing cell, supporting this notion and validating our approach. The tension around a dividing cell was previously found to change by ≈ 10–20% in a distance of up to two cell diameters from the division event, being similar to our estimated effect size of 2–3 cell sizes^54^. Under certain conditions, proliferation events are sufficient and/or necessary for the induction of cellular reorganization and a migratory phenotype^17,27,30^. We could confirm an effect of proliferation events on the local velocity field, spanning a similar spatial scale as in MDCK cells^18^. Although, here the effect was small on the tissue scale. A potential reason for this discrepancy might lie in the cellular system used. Here, highly motile GB cells were used moving between 10 and 18 μm/h, while in the aforementioned studies cells with lower migratory capacity were investigated. In a very similar setup MDCK cells moved at most ≈ 6 μm/h^30^, implying a significantly lower cell-intrinsic force generation, helping to overcome cell density induced confinement. To the authors’ knowledge, there is only one study on mamma carcinoma cells evaluating the impact of proliferation events on tumor cell migration^55^. West et al. reported similar speeds for the monolayer as observed here, and found the effect of proliferation events to drop off in a distance of one cell diameter, with a similar magnitude of the effect. Albeit similar, the effective range was lower, potentially due to different biological behavior of the used tumor entities or the significantly lower number of analyzed division events. Thus, most results obtained here are in good agreement with earlier studies, showing a local effect of proliferation events on the velocity field, but here cell divisions were not the main driver of migration, likely due to the higher intrinsic cell mobility of GB cells. Interestingly, the cells with the highest proliferation rates in our study (LN229, GBM10) were the least migratory for high cell densities, which agrees with previous studies demonstrating a negative correlation between proliferation and migration^56,57^.

The second key aspect in the present study was the effect of cell density gradients on GB migration, a situation cells potentially face when reaching the tumor-stroma boundary, long distance fiber tracts or the perivascular space. In case of GB, tumor borders are not clearly defined, but a decreasing cell density with distance from the main tumor mass, together with varying cell densities inside the tumors were reported^58^. When subjecting cells to a large-scale cell density gradient, two main factors were expected to drive the expansion of the cell clusters, namely the active forces generated by cells, transmitted via cell-cell junctions and a passive expansion driven by increasing pressure due to proliferation^59^. A previous study by Tlili et al. suggested if proliferation and thus stress gradients are the main drivers of expansion, spreading is expected to have characteristics of a non-active material wetting a substrate. Then, velocity and density profiles depend on the distance to the front^59^. Blocking of proliferation led to a cell expansion pattern determined by cell density and cell autonomous processes, while for high proliferation rates the velocity was mostly governed by cell division^59^. Given the results for LN229 and GBM10 cells, both cell types are highly proliferative, with velocity and cell density being correlated with the distance to the cell front. Vanishing those gradients, achieved via the blockade of proliferation, led to a stop of expansion. Consequently, both cell lines likely expanded in a passive way driven by proliferation, mostly independent of cell-autonomous processes. In U138 cells no consistent cell-density gradient was observed during cluster expansion, independent of proliferation, implying expansion was driven by active cell migration. An in silico study predicted a growth driven cellular expansion, if the cells have a high proliferation rate but low mobility^60^. Given the determined cell division numbers and single cell migration properties, LN229 and GBM10 cells were more proliferative and less migratory than U138 and GBM4 on the single cell level, supporting the idea of a mostly passive expansion of LN229 and GBM10.

In the last part of this study, the effect of increasing cell density was analyzed. In line with the results from single cell migration and cell exclusion experiments, LN229 and GBM10 decreased their effective mobility in terms of the MSD and scaling coefficient more than U138 and GBM4, with increasing cell densities. One reason for this phenomenon might be their different migratory capacity on the level of individual cells with lower mobility for LN229 and GBM10. Thus, high cell densities were more likely to impede their movement. However, under this assumption, an impeded cell-body translocation and a less super-diffusive movement of U138 and GBM4 cells would be expected too, but especially the super-diffusive movement type remained almost unaffected. The slowdown and potential migratory arrest of LN229 and GBM10 for high cell densities are in agreement with the expectations from the literature^6–13,16^, arguing that cell density can regulate traction forces^10,13^. Cell density independence of collective migration was reported less frequently, e.g. in a study of mammary carcinoma cells^48^ and a previous study of ours in GB^12^. For the more motile cells a parallel alignment of cells, combined with a movement in alignment-direction as anti-parallel streams was observed. Such streaming behavior was found to be favored under conditions of low contractility, high mobility and high cell density^15,32^. These findings provide a potential explanation for the phenotypical differences observed here, as we previously reported LN229 to be more contractile but similarly adhesive as U138^12^. Furthermore, LN229 and GBM10 were here less motile on the single cell level.

The observed structures formed by GB in vitro resembled those of oncostreams as previously identified in vivo, promoting collective GB migration^34^. Yet, an in silico study predicted anti-parallel oncostreams, as observed here, to be unstable and transform into a parallel streams over the time frame of days^32^. We could not observe a shift in movement pattern, but found a strengthening of the antiparallel streaming over time. However, high cell density and cell speeds were proposed as factors blocking or delaying the switch from anti-parallel to parallel streaming^32^. Given the modeling parameters used, the stability of the anti-parallel streams could be expected for the self-organizing cell lines. Of note, those oncostreams were found to resemble the topology of liquid crystals with a quasi-long-range order and similar order parameter values as observed here^35^. For higher cell densities, an increase in the nematic order was found for GBM4 and U138, accompanied by the expected decrease in the number of topological defects, due to defect annihilation^61–63^. In contrast to other studies, a favored direction of movement of topological defects relative to their orientation was absent here, so they were neither extensile nor contractile^62,64,65^. However, the overall movement range of the defects appeared to be similar as in epithelial cells^65^. Independent of the properties of the nematic defects, organization into the liquid crystal-like structure seemed to be associated with the escape from cell density induced migratory arrest. This interpretation was strengthened by the observation that abrogating the formation of oncostreams blocks tumor infiltration and migration in vivo^34^. Furthermore, if liquid crystal-like organization is correlated with sustained migration under confinement, then locally organized clusters are expected to migrate more effective. This situation corresponds to a motile group of cells inside a group of immobile cells. An in silico study predicted for this case an impeded flow eventually leading to the formation of organized, more mobile clusters inside the immobile cell population^32^. This prediction corresponded to the observations made in LN229 cells here, supporting the notion of self-organization to be associated with migration. Interestingly, in other tumor entities self-organization may play a role in invasion. In mamma carcinoma cells invasive tumor cells were proposed to move through the tissue as active nematic aggregates^36^. In general, collective tumor invasion happens amongst others in form of invading cell chains, or collective strands, accompanied by a certain polar alignment of cells in such protrusions to allow for effective invasion^66^. Thus, limited self-organization of tumor cells could potentially be a more general feature involved in migration and invasion. However, causality between liquid crystal-like cellular organization and sustained cell migration is not yet established and future studies actively inducing or disturbing cellular self-organization are needed. Furthermore, many questions remain unanswered, including the molecular basis for the self-organization and sustained mobility. Other questions include the role of the tumor microenvironment, such as astrocytes that can account for a relevant number of cells inside a tumor mass, and surround GB in the form of reactive astrocytes. Those cells form obstacles inside the tumor mass, but also change their intracellular signaling.

In conclusion, this study demonstrated the existence of two migratory distinct phenotypes of GB cells, one in which cell density reduces effective motion and another that stays comparably mobile for high cell densities, but shows strong signs of self-organization. These findings suggest that liquid crystal-like organization may be an important correlate of sustained GB cell motility under high-density conditions and provides a framework for studying how collective organization may contribute to GB invasion.

## Supplementary Information

Below is the link to the electronic supplementary material.

Supplementary Material 1

Supplementary Material 2

Supplementary Material 3

Supplementary Material 4

Supplementary Material 5

Supplementary Material 6

Supplementary Material 7

Supplementary Material 8

Supplementary Material 9

Supplementary Material 10

## Data Availability

All data generated or analysed during this study are included in this published article (and its Supplementary Information files).
