# Supplementary material for: Liquid crystal-like self-organization of glioblastoma is associated with consistent migration for high cell densities
Source: Sci Rep. 2026 Jun 22;16:19339. doi: 10.1038/s41598-026-58846-8 (PMC13287651; doi:10.1038/s41598-026-58846-8)
Supplement: Supplementary file 1 — Supplementary Material 1 [file 41598_2026_58846_MOESM10_ESM.docx]

**Liquid Crystal-like Self-Organization of Glioblastoma Prevents Cell Density Induced Migratory Arrest**

Urszula Hohmann^1^, Chalid Ghadban^1^, Julian Prell^2^, Christian Strauss^2^, Faramarz Dehghani^1^, Tim Hohmann^1,*^

1) Department of Anatomy and Cell Biology, Medical Faculty, Martin Luther University Halle-Wittenberg, 06108 Halle (Saale), Germany

2) Department of Neurosurgery, Medical Faculty, Martin Luther University Halle-Wittenberg, 06120 Halle (Saale), Germany

**Supplementary Figures:**


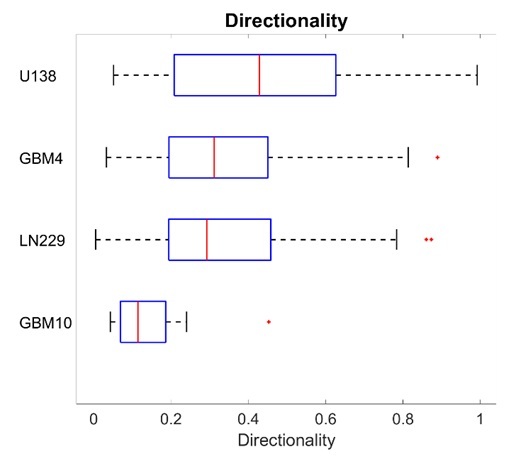


**Supplementary Figure 1:** Directionality of individual GBM cells. Box plots show the median (red line), 25 and 75 percentile (box), non-outlier range (whiskers) and outliers (red dots). Sample sizes: n_U138_ = 44; n_GBM4_ = 47; n_LN229_ = 83; n_GBM10_ = 43;


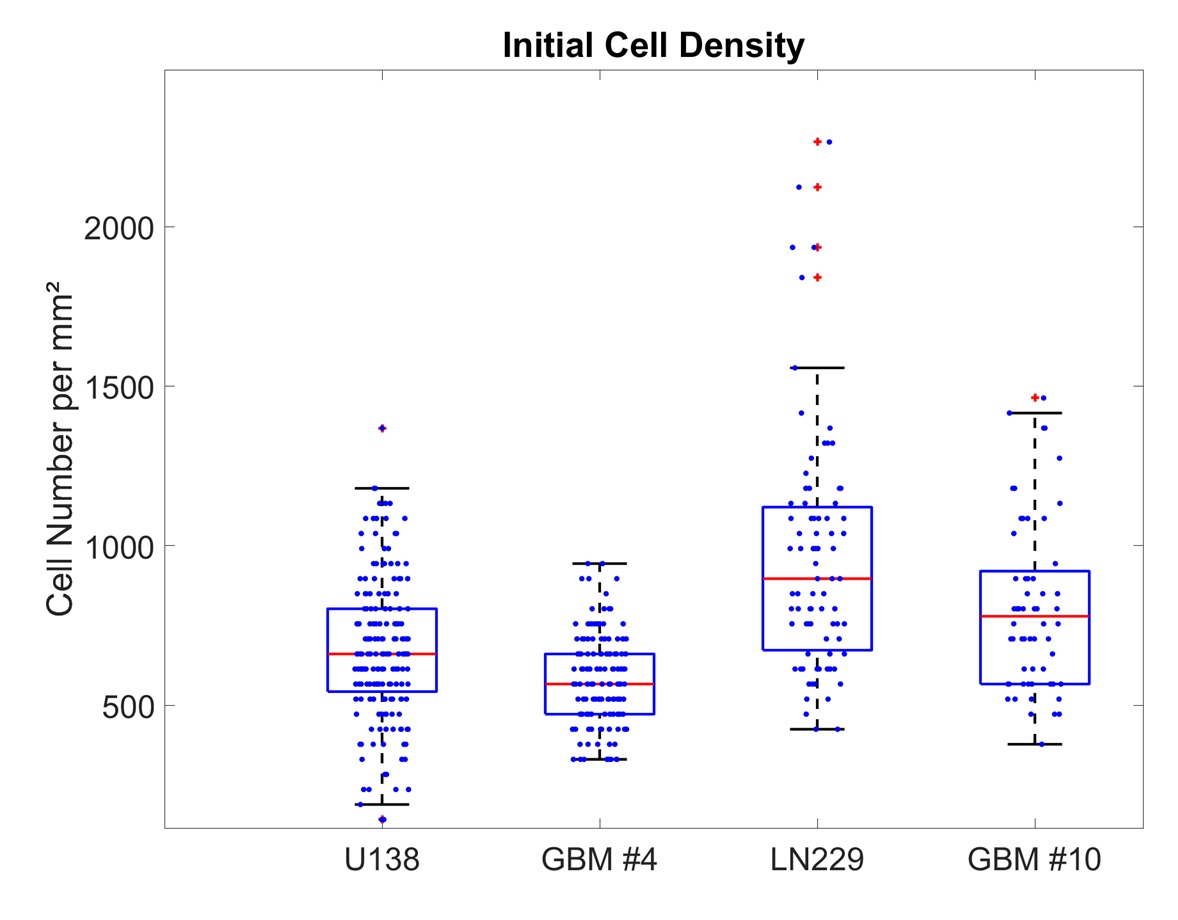


**Supplementary Figure 2:** Manual estimates of the initial cell density at 0 h for all GBM cell lines. Box plots show the median (red line), 25 and 75 percentile (box), non-outlier range (whiskers) and outliers (red dots). Sample sizes: n_U138_ = 184; n_GBM4_ = 139; n_LN229_ = 79; n_GBM10_ = 60;


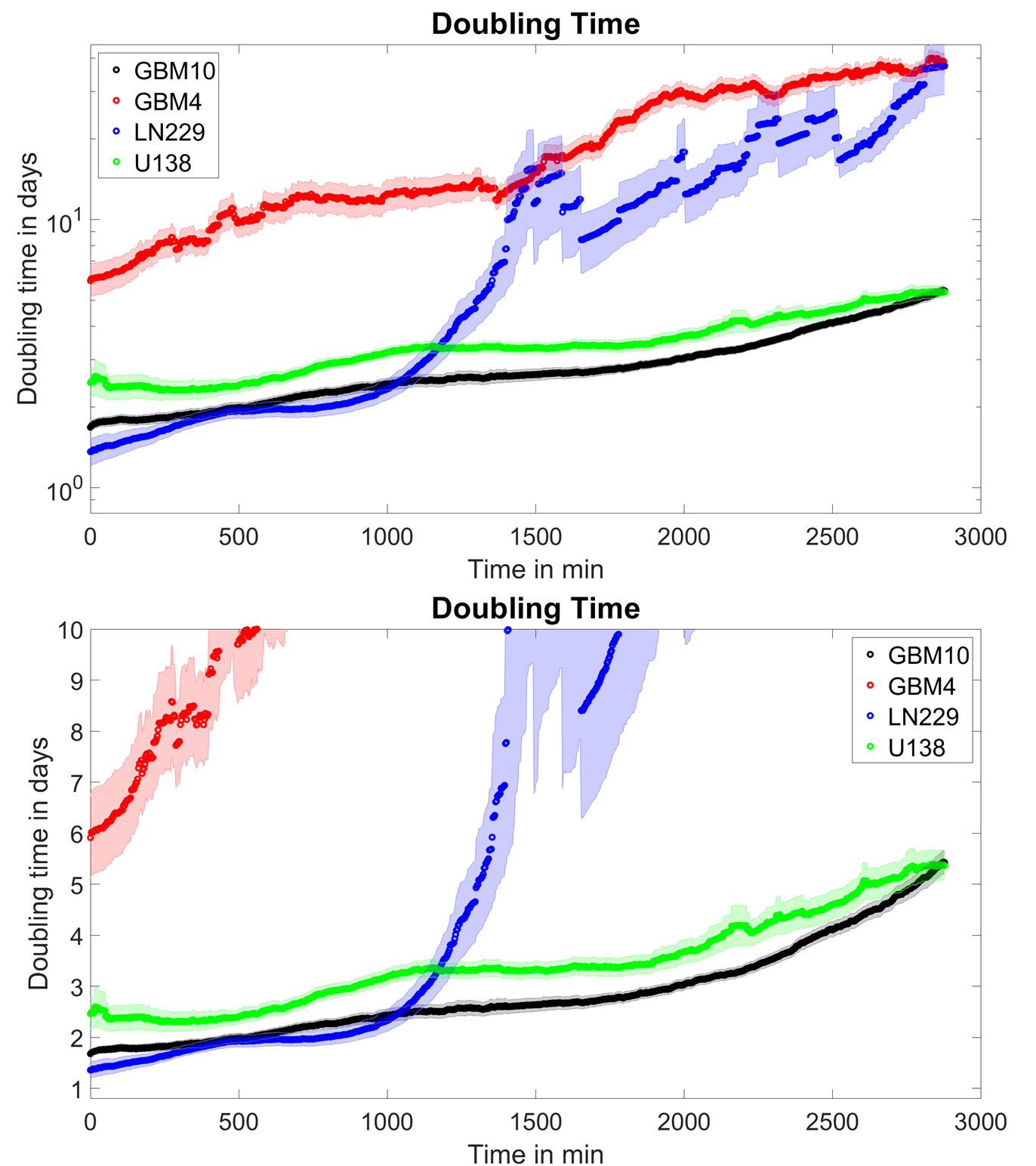


**Supplementary Figure 3:** Doubling time for GBM cells in a confluent monolayer as a function of measurement time. Top: Log-y plot. Bottom: Linear scale.


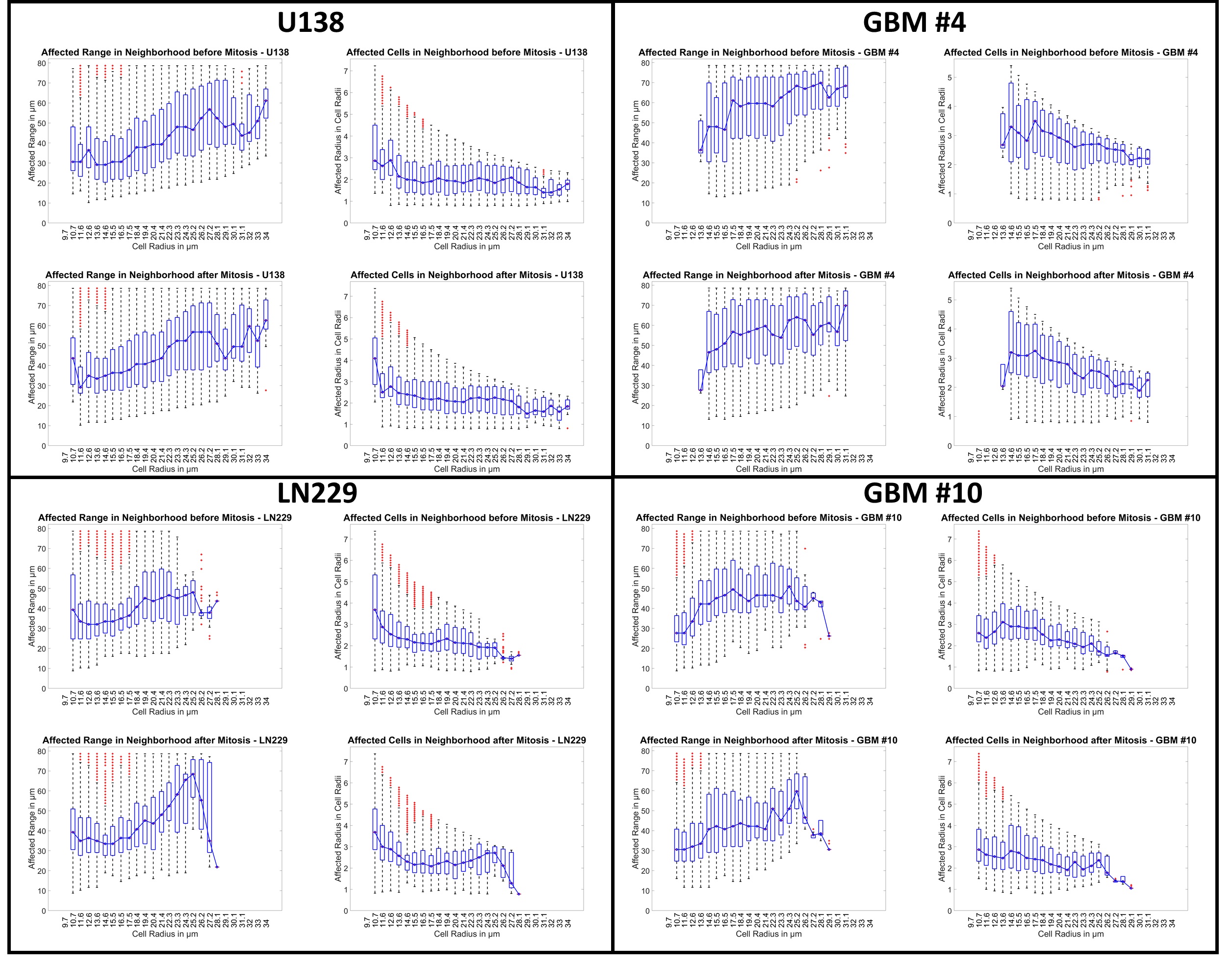


**Supplementary Figure 4:** Plots of the effect range of a proliferation event on the velocity field as a function of cell density. For each of the four cell lines a plot of the affected range by pre-mitotic contraction in µm (top left) and cell length (top right), as well as the affected range by post-mitotic expansion in µm (bottom left) and cell length (bottom right) is given as a function of effective cell radius. Box plots show the median (red line), 25 and 75 percentile (box), non-outlier range (whiskers) and outliers (red dots). Each boxplot contains at least 70 division events.


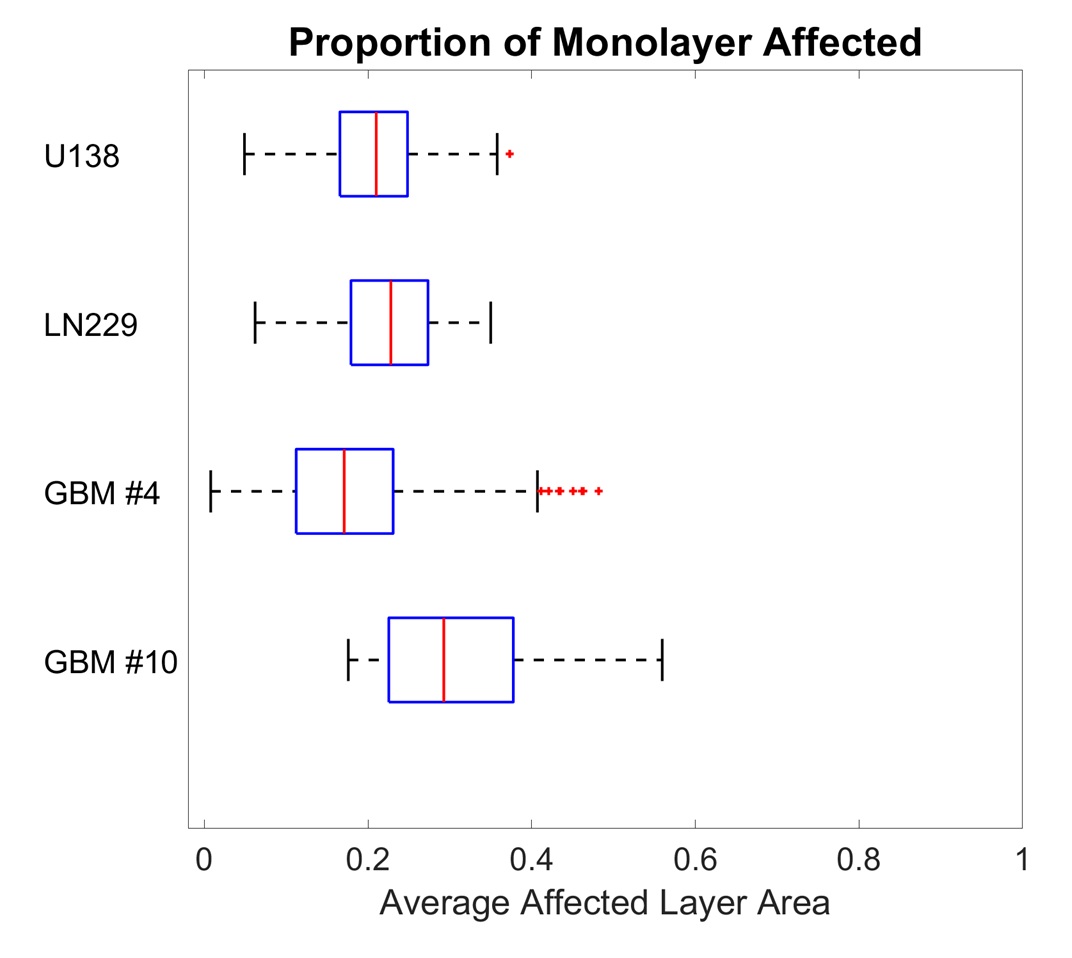


**Supplementary Figure 5:** Boxplot of the relative area of the monolayer in which the velocity field is affected by proliferation events. Box plots show the median (red line), 25 and 75 percentile (box), non-outlier range (whiskers) and outliers (red dots). Sample sizes: n_U138_ = 184; n_GBM4_ = 139; n_LN229_ = 79; n_GBM10_ = 60;


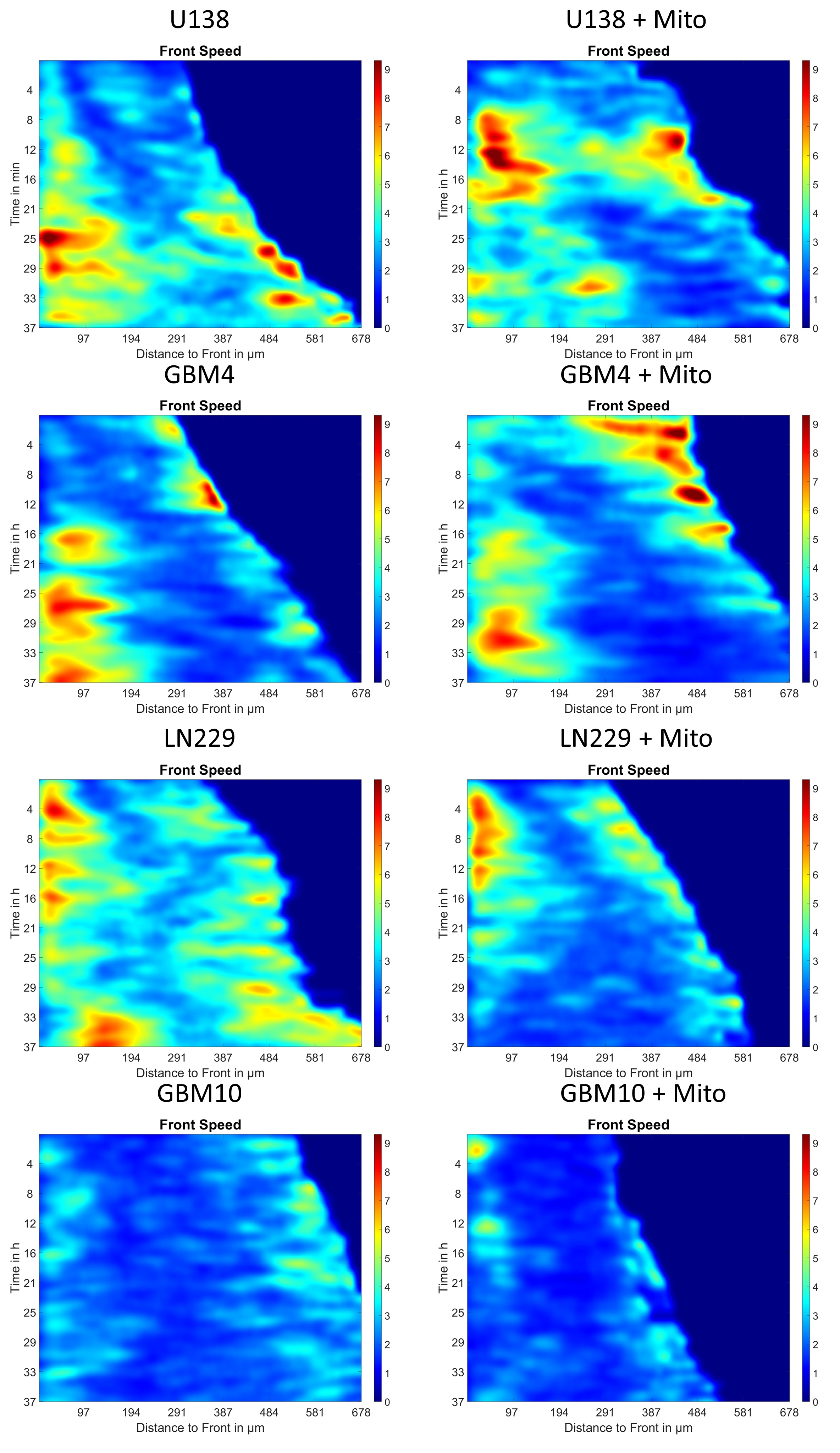


**Supplementary Figure 6:** Sample heatmaps of front speeds for the cell exclusion assay. The figure shows examples of the layer speed as a function of time and distance to the cell front for all four GBM cell lines, under control conditions (left column) and when treated with mitomycin C (right column).


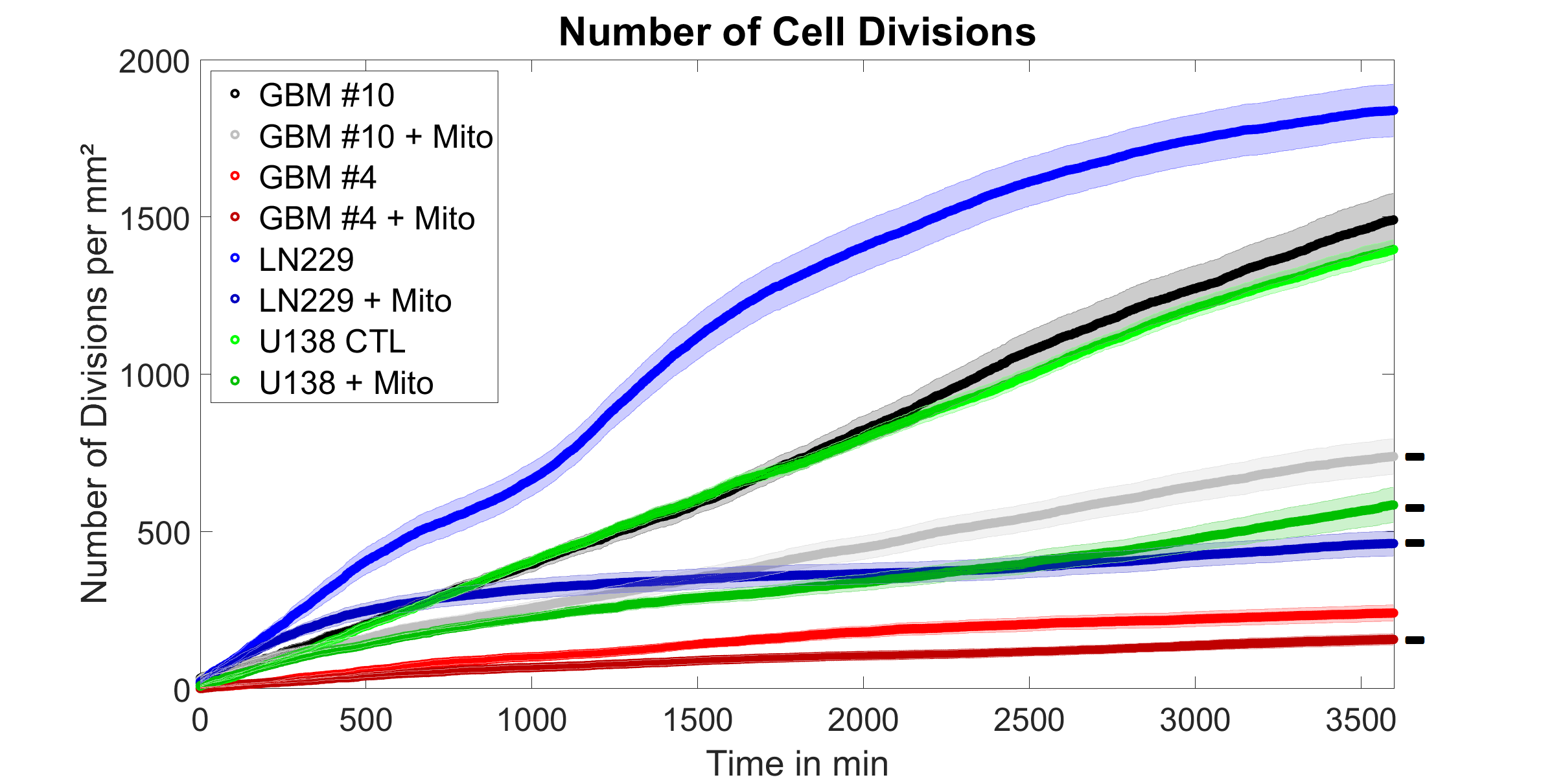


**Supplementary Figure 7:** Number of cell divisions in absence or presence of mitomycin C. The “-“ sign corresponds to groups with a significant reduction in the number of proliferation events at the end of the measurement period of the mitomycin C treated group, relative to the respective untreated control with p<0.05. P values were calculated using a two-sided sign test. Sample Sizes: n_U138 CTL_ = 24; n_U138 Mito_ = 11; n_GBM4 CTL_ = 39; n_GBM4 Mito_ = 10; n_LN229 CTL_ = 29; n_LN229 Mito_ = 15; n_GBM10 CTL_ = 13; n_GBM10 Mito_ = 13


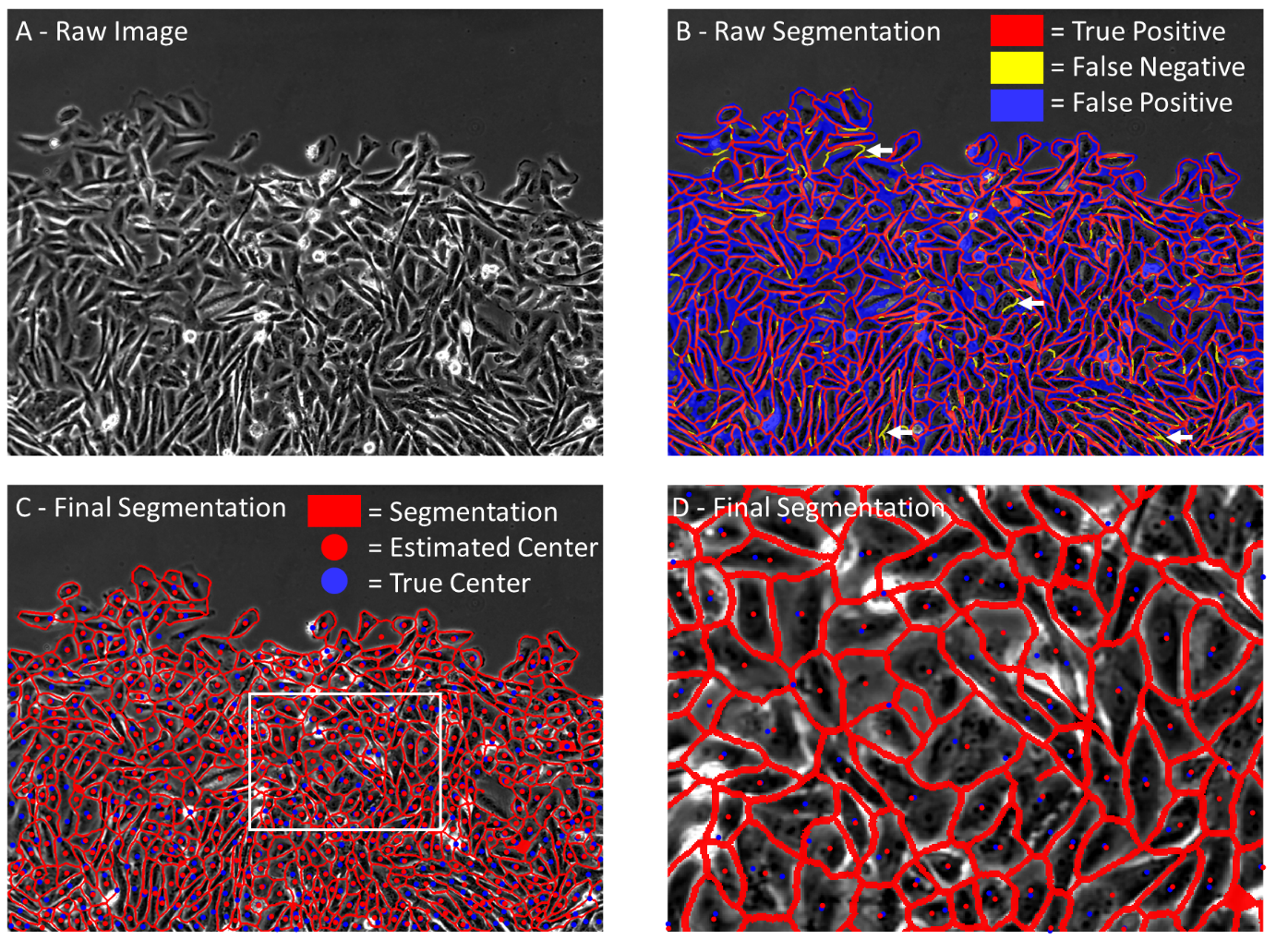


**Supplementary Figure 8:** Illustration of the segmentation of the monolayer in the cell exclusion assay. A) Raw sample image of the test data set. B) Raw segmentation of the sample image of A) after application of the U-Net without further post-processing. Red denotes true positive, yellow false negative and blue false positive pixels. All other pixels are true negatives. White arrows point to some cell-cell edges that have not been fully detected (false negatives). C) Final segmentation of the image in A), after initial U-Net segmentation and post-processing. Red lines correspond to detected cell edges. Red dots show the center of the segmented cells, while blue dots show the corresponding ground truth centers. The white rectangle shows the area that is enlarged in D).


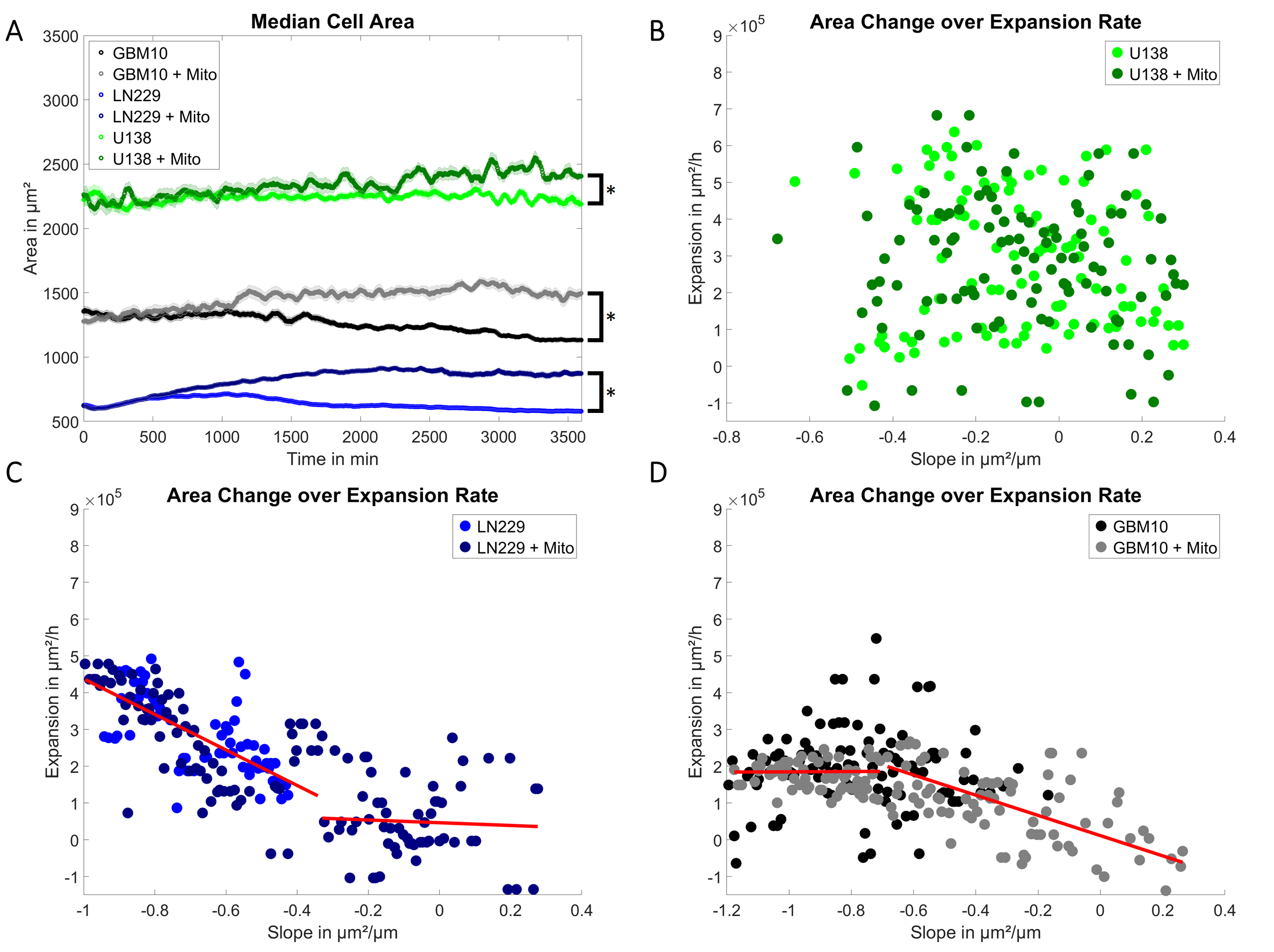


**Supplementary Figure 9:** Relation of cell size and expansion rate in cell exclusion assay. A) Graph of the time evolution of average cell size in the field of view as a function of time under control conditions and when treated with mitomycin C. B) to D) Scatter plots of the expansion rate of the layer over the cell density gradient in the layer for U138 cells (B), LN229 (C) and GBM10 (D) cells. Red lines show linear fits to the two different regimes found in C) and D). Error bars and shaded areas depict the standard error of the mean. Stars depict significant differences between the last time point of groups with p<0.05 calculated using a two-tailed ANOVA with the Tukey post-hoc test. Sample Sizes: A) n_U138 CTL_ = 24; n_U138 Mito_ = 11; n_LN229 CTL_ = 29; n_LN229 Mito_ = 15; n_GBM10 CTL_ = 13; n_GBM10 Mito_ = 13 B) n_U138 CTL_ = 24; n_U138 Mito_ = 11 C) n_LN229 CTL_ = 29; n_LN229 Mito_ = 15 D) n_GBM10 CTL_ = 13; n_GBM10 Mito_ = 13.


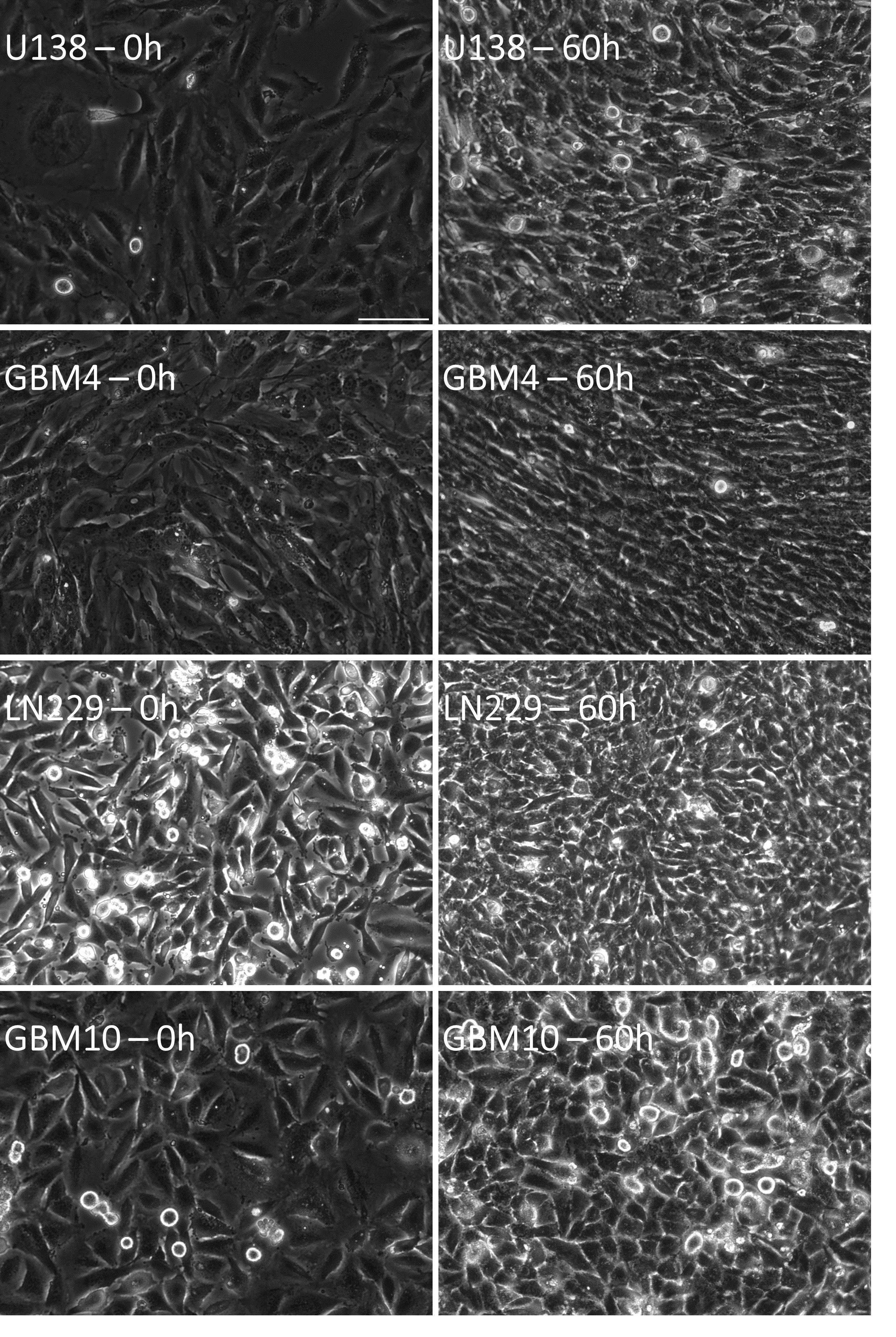


**Supplementary Figure 10:** Sample images showing different cellular organizations for the four GBM cell lines, at the start of the experiments (left) and after 60 h (right). Please note the strong emerging order in U138 and GBM4 cells. Scale bar corresponds to 100 µm.


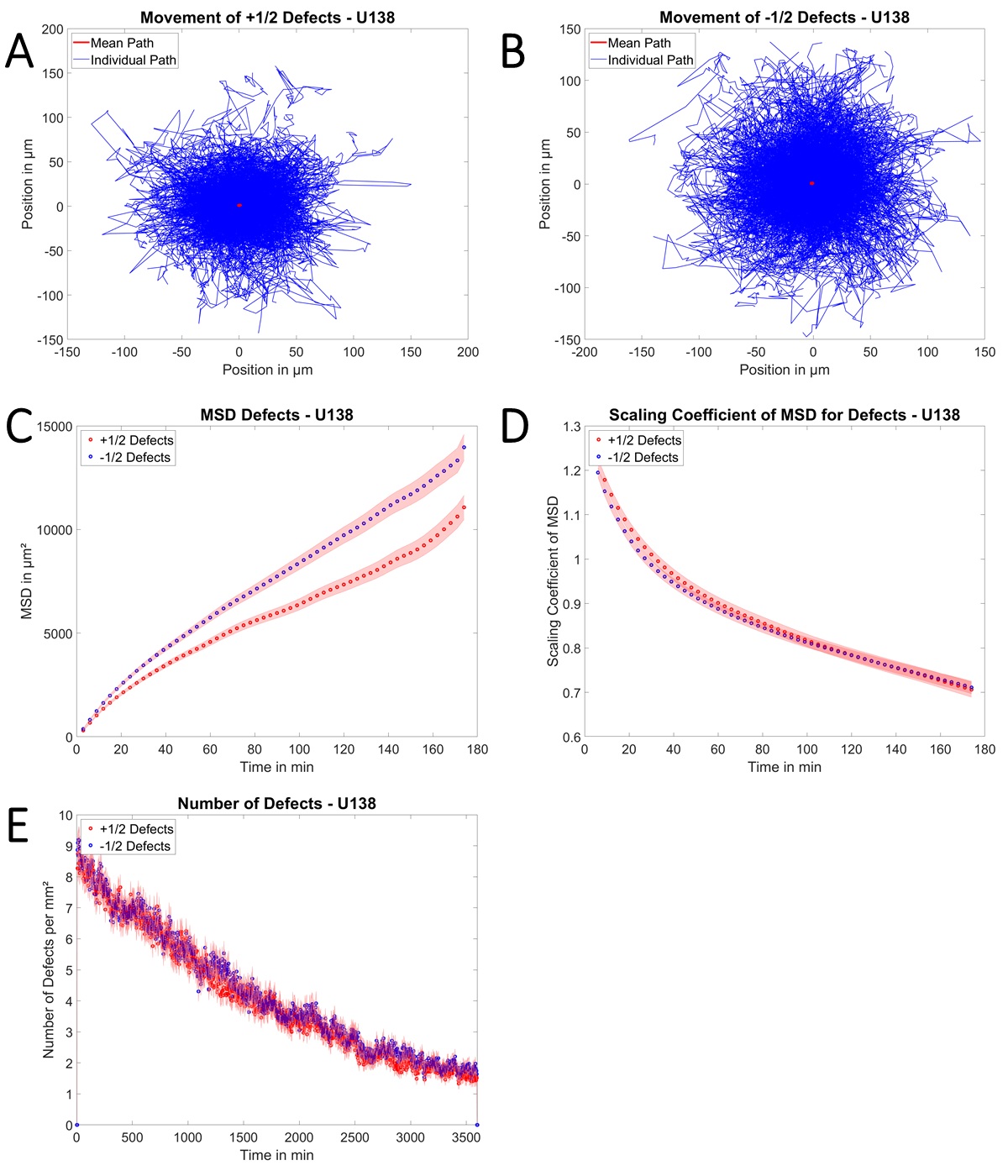


**Supplementary Figure 11:** Analysis of topological defects in U138 cells. A), B) Movement paths of +1/2 (A) and -1/2 (B) defects relative to their orientation. The red line shows the mean movement path over all defects. C), D) Mean squared displacement (C) and the associated scaling coefficient (D) for defects. E) Temporal evolution of the number of defects. Error bars and shaded areas depict the standard error of the mean. Sample size: A)-D) n_+1/2_ = 535; n_-1/2_ = 703; E) n = 184.


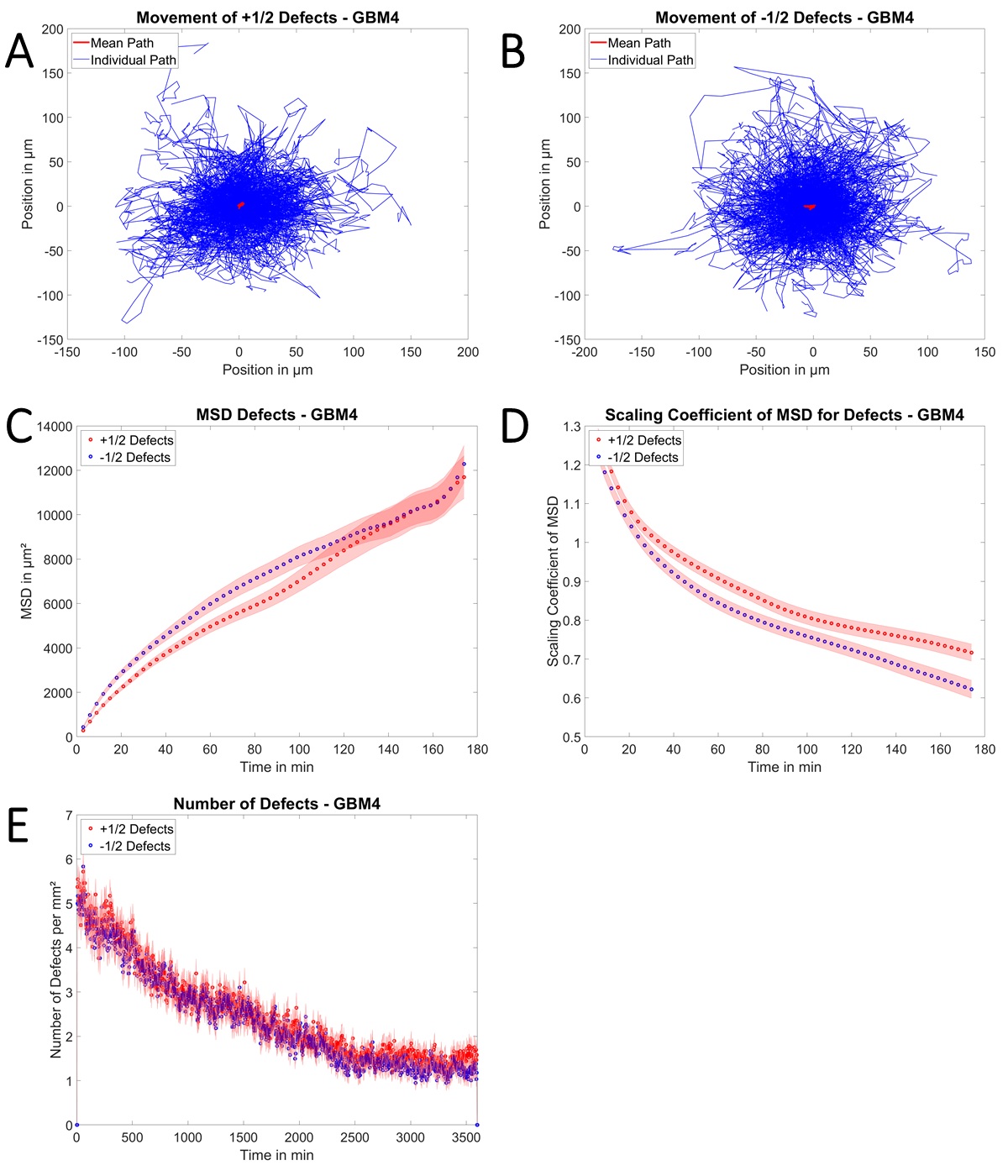


**Supplementary Figure 12:** Analysis of topological defects in GBM4 cells. A), B) Movement paths of +1/2 (A) and -1/2 (B) defects relative to their orientation. The red line shows the mean movement path over all defects. C), D) Mean squared displacement (C) and the associated scaling coefficient (D) for defects. E) Temporal evolution of the number of defects. Error bars and shaded areas depict the standard error of the mean. Sample size: A)-D) n_+1/2_ = 279; n_-1/2_ = 286; E) n = 139.


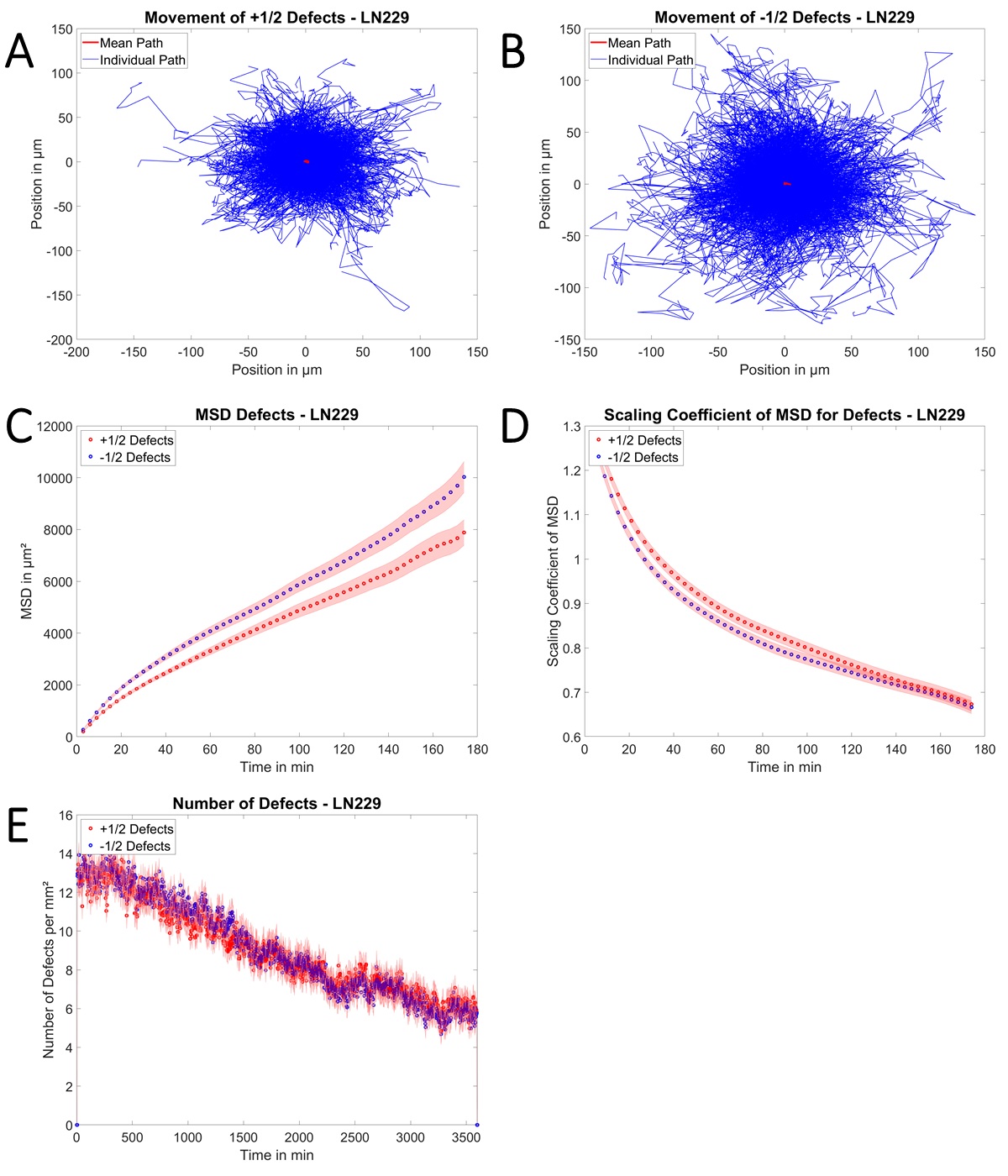


**Supplementary Figure 13:** Analysis of topological defects in LN229 cells. A), B) Movement paths of +1/2 (A) and -1/2 (B) defects relative to their orientation. The red line shows the mean movement path over all defects. C), D) Mean squared displacement (C) and the associated scaling coefficient (D) for defects. E) Temporal evolution of the number of defects. Error bars and shaded areas depict the standard error of the mean. Sample size: A)-D) n_+1/2_ = 603; n_-1/2_ = 685; E) n = 79.


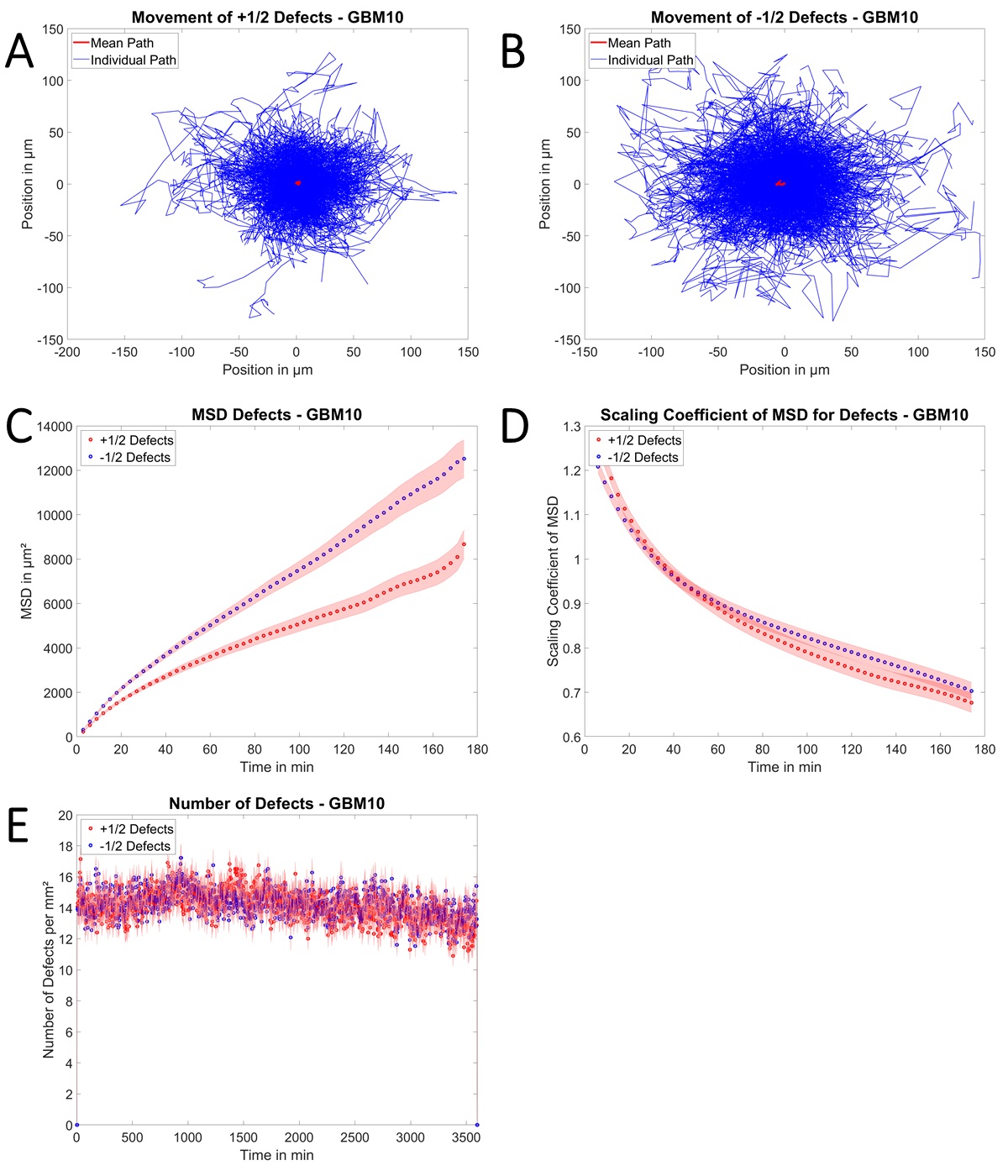


**Supplementary Figure 14:** Analysis of topological defects in GBM10 cells. A), B) Movement paths of +1/2 (A) and -1/2 (B) defects relative to their orientation. The red line shows the mean movement path over all defects. C), D) Mean squared displacement (C) and the associated scaling coefficient (D) for defects. E) Temporal evolution of the number of defects. Error bars and shaded areas depict the standard error of the mean. Sample size: A)-D) n_+1/2_ = 298; n_-1/2_ = 419; E) n = 60.


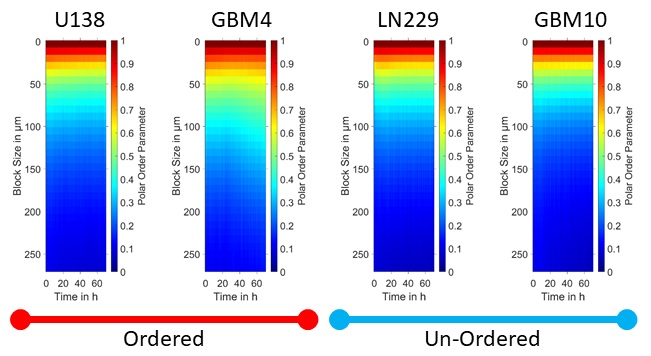


**Supplementary Figure 15:** Heatmaps of the temporal and spatial evolution of the polar order parameter calculated for the velocity field. Sample sizes: n_U138_ = 184; n_GBM4_ = 139; n_LN229_ = 79; n_GBM10_ = 60.


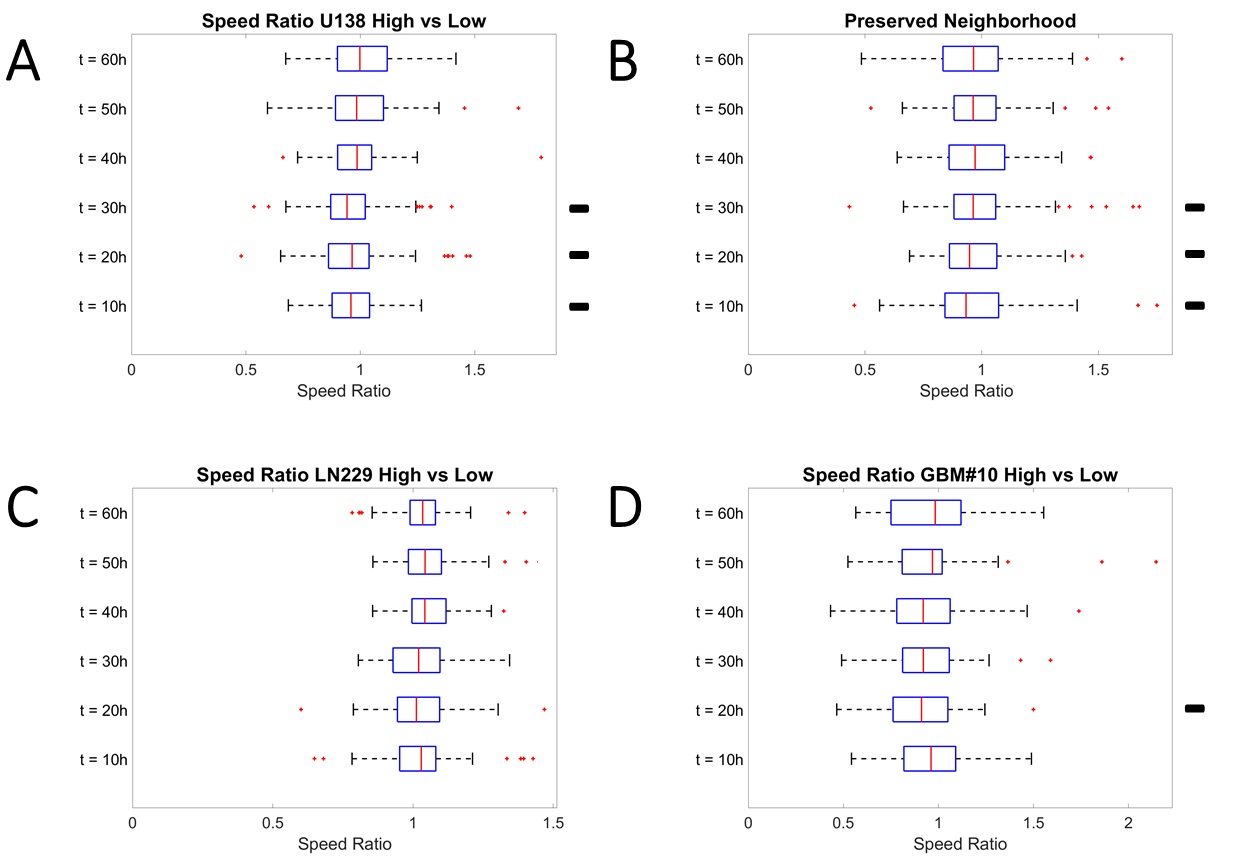


**Supplementary Figure 16:** Comparison of the speed of ordered and unordered regions in one field of view. A) to D) Ratios of the speed for regions with high nematic order over those with low nematic order for U138 (A), GBM4 (B), LN229 (C) and GBM10 (D). The “-“ sign corresponds to groups with a significant reduction in speed (p<0.05) as determined by the two-sided sign test. Box plots show the median (red line), 25 and 75 percentile (box), non-outlier range (whiskers) and outliers (red dots). Sample sizes for t = 0,10,20,30,40,50,60h: B): n_U138_ = 180, 167, 161, 145, 135, 101; n_GBM4_ = 139, 131, 122, 115, 106, 88; n_LN229_ = 79, 79, 78, 78, 77, 73; n_GBM10_ = 32, 27, 28, 29, 28, 21.

Supplementary Table 1:

| Cell Line | # ROIs | # Divisions |
| --- | --- | --- |
| GBM #10 | 60 | 24,612 |
| GBM #4 | 139 | 16,645 |
| LN229 | 79 | 34,993 |
| U138 | 184 | 58,131 |
| Sum | 462 | 134,381 |

**Supplementary Table 1:** Number of detected cell division events and number of analyzed fields of view for each cell line.

Supplementary Table 2:

|  | True Positive | False Positive | False Negative | True Negative | F1-Score | Accuracy |
| --- | --- | --- | --- | --- | --- | --- |
| Pixel-wise | 19.4% | 13.4% | 2% | 65.2% | 0.70 | 0.85 |
| Cell-wise | 65.0% | 10.2% | 24.8% | --- | 0.77 | --- |

**Supplementary Table 2:** Confusion matrix and measures of goodness for the cell-layer segmentation approach used for the cell-exclusion assay.

Supplementary Table 3:

|  | First regime | | | Second regime | | |
| --- | --- | --- | --- | --- | --- | --- |
|  | Slope  [10^5^ µm/h] | Intercept [10^5^ µm²/h] | p-Value against constant | Slope  [10^5^ µm/h] | Intercept [10^5^ µm²/h] | p-Value against constant |
| U138 | 2,76 | -0.82 | 0.178 | --- | --- | --- |
| LN229 | -4.83 | -0.46 | <10^-9^ | -0.37 | 0.46 | 0.592 |
| GBM10 | 0.04 | 1,88 | 0.634 | -2.70 | 0.10 | <10^-9^ |

**Supplementary Table 3:** Fit parameters obtained for the analysis of cell layer expansion rate as a function of the cell density gradient.

**Supplementary Video 1:** Cell exclusion assay for GBM4 cells, showing expansion and signs of self-organization.

**Supplementary Video 2:** Cell exclusion assay for U138 cells, showing expansion and signs of self-organization.

**Supplementary Video 3:** Cell exclusion assay for GBM10 cells, showing expansion and no signs of self-organization.

**Supplementary Video 4:** Cell exclusion assay for LN229 cells, showing expansion and no signs of self-organization.

**Supplementary Video 5:** Cell exclusion assay for LN229 cells treated with mitomycin C, showing expansion and no signs of self-organization.

**Supplementary Video 6:** Confluent monolayer for GBM4 cells, showing migration and emergence of anti-parallel moving streams and self-organization.

**Supplementary Video 7:** Confluent monolayer for U138 cells, showing migration and emergence of anti-parallel moving streams and self-organization.

**Supplementary Video 8:** Confluent monolayer for GBM10 cells, showing little migration and no emergence of anti-parallel moving streams or self-organization.

**Supplementary Video 9:** Confluent monolayer for LN229 cells, showing migration and no large-scale emergence of anti-parallel moving streams or self-organization.
